# Emerging Nanonutraceuticals against Obesity

**DOI:** 10.1002/smsc.202500116

**Published:** 2025-07-22

**Authors:** Linjie Ni, Minmin Peng, Yiying Liang, Xiwen Ye, Hanying Zheng, Dongbei Guo, Liang Yang, Xusangni Li, Ronghe Chen

**Affiliations:** ^1^ State Key Laboratory of Vaccines for Infectious Diseases Xiang An Biomedicine Laboratory Xiang'an Hospital of Xiamen University National Innovation Platform for Industry‐Education Integration in Vaccine Research School of Public Health Xiamen University Xiamen 361102 China; ^2^ Department of Clinical Laboratory Nanan Hospital Quanzhou 362300 China; ^3^ Department of Nutrition 900th Hospital of PLA Joint Logistic Support Force Fuzhou 350025 China

**Keywords:** bioavailability, nanonutraceuticals, obesity

## Abstract

Obesity, a global public health issue, often emanates from dietary imbalances. Strategic nutraceutical supplementation can fundamentally mitigate or even reverse obesity with minimal adverse effects. Nonetheless, most antiobesity nutraceuticals possess intrinsic limitations in oral administration, such as low solubility, chemical instability, and susceptibility to biodegradation. These drastically diminish their bioavailability, thereby restricting their effectiveness in combating obesity. The emergence of nanotechnology heralds a transformative paradigm to clear this hurdle. A myriad of nutraceuticals has been nanomodified for obesity management, yet a comprehensive compilation on this topic is currently absent. Accordingly, this review focuses on all antiobesity nanonutraceuticals and expounds their characteristics, enhanced bioavailability, and weight‐loss mechanisms according to six primary categories: carbohydrates, lipids, proteins, vitamins, minerals, and phytochemicals. Their application perspectives, impediments, and potential solutions are further discussed. As a pivotal reference, this review will accelerate the in‐depth development of nanonutraceuticals and their translation from bench to bedside in the ongoing battle against the global obesity epidemic.

## Graphical abstract

This overview comprehensively compiles the latest research advancements in diversiform nanonutraceuticals against obesity. These nanonutraceuticals are derived from the nanomodifications of some carbohydrates, lipids, proteins, vitamins, minerals, and phytochemicals. Their characteristics, bioavailability, antiobesity effectiveness, and application perspectives and challenges are highlighted to serve as a reference for the comprehensive development and clinical translation of advanced nanonutraceuticals for weight loss.

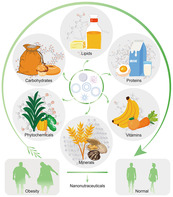

## 1 Introduction

Obesity is a pathological condition resulting from an imbalance between caloric intake and energy expenditure. According to the World Health Organization, ≈12.5% of the global population (over 1 billion people) were obese in 2022.^[^ ^1^ ^]^ Since 1990, obesity has more than doubled in adults and quadrupled in adolescents. If this trend continues, the number of obese individuals aged 25 years and older will rise to 1.95 billion by 2050.^[^ ^2^ ^]^ Obesity is a major contributor to the development of type 2 diabetes, cardiovascular diseases, cancer, liver diseases, and other disorders.^[^ ^3^ ^]^ These diseases notably increase the proportion of people needing healthcare, which escalates the financial burden on hospitals and insurance companies. By 2035, the financial burden associated with overweight and obesity is expected to exceed $4 trillion.^[^ ^4^ ^]^ Confronted with such dire consequences, several therapeutic strategies have been proposed to curb obesity, including lifestyle interventions, pharmacological treatments, and surgery.^[^ ^5^, ^6^, ^7^ ^]^ Although improvements are observed in numerous cases, the predominant challenges remain side effects and low compliance.

Nowadays, a popular, relatively safe, and cost‐effective antiobesity approach involves taking nutraceuticals that offer both nutritional and pharmacological benefits, such as dietary fibers, fatty acids, bioactive proteins, vitamins, minerals, and phytochemicals. They can inhibit caloric intake and enhance energy expenditure through various biological pathways^[^ ^6^, ^8^, ^9^ ^]^ to achieve effective obesity control. Nevertheless, these nutraceuticals have inherent deficiencies like poor solubility, chemical instability, unpleasant odor, and bitter taste. In the alimentary tract, they are susceptible to degradation by gastrointestinal membrane barriers, violent acid–base fluctuations, reactions with digestive enzymes, and food interference.^[^ ^10^ ^]^ Inefficient intestinal epithelium permeation also affects their effective dosage into the circulation.^[^ ^11^ ^]^ These factors ultimately result in poor bioavailability of nutraceuticals when administered orally, which severely constrains their capacity to combat obesity. Hence, there is an urgent need to overcome this predicament.

Nanotechnology is an emerging field of multidisciplinary research on 1–100 nm particulates. Utilizing this technology, nutraceuticals can be transformed into diverse nanosized forms, such as polymeric nanoparticles (NP), nanocrystals, micelles, liposomes, niosomes, nanoemulsions, and inorganic NP.^[^ ^12^, ^13^ ^]^ Nanonutraceuticals exhibit improved physiochemical properties including surface area, stability, solubility, biocompatibility, and nutritional properties.^[^ ^14^, ^15^, ^16^, ^17^ ^]^ Due to their small size and high surface area, nanonutraceuticals are easily internalized by cells. Moreover, with surface modifications or encapsulation techniques, they can survive in different parts of the gastrointestinal tract and be delivered to specific sites without affecting normal cells in the surrounding area.^[^ ^18^, ^19^, ^20^ ^]^ It is evident that the advent of nanotechnology offers novel solutions for improving the oral bioavailability of certain nutraceuticals. Although a multitude of nutraceuticals have been nanomodified to enhance their antiobesity efficacy, there is a paucity of reviews that have attempted to compile these studies comprehensively.

**Figure 1:**
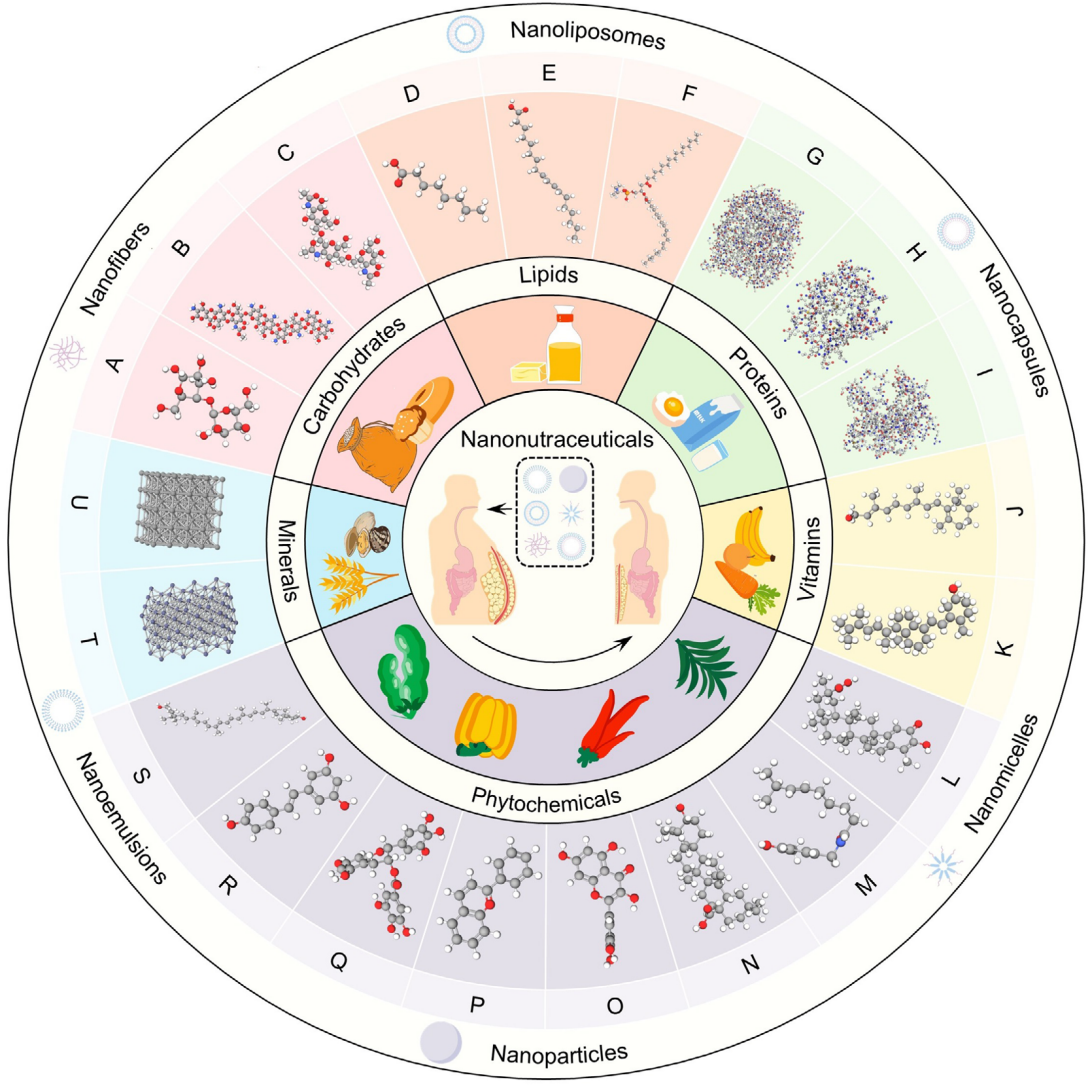
Overview of diversiform nanonutraceuticals against obesity. These complexes are derived from nanomodifications of carbohydrates (e.g., A) cellulose, B) chitosan, and C) chitin), lipids (e.g., D) MCFA, E) CLA, and F) PPC), proteins (e.g., G) GL protein, H) Cyt *c*, and I) TTI), vitamins (e.g., J) vitamin A and K) vitamin (D), phytochemicals (e.g., L) CLT, M) capsaicin, N) OA, O) quercetin, P) anthocyanins, Q) EGCG, R) resveratrol, and S) lutein), and minerals (e.g., T) zinc and U) chromium).

Herein, from a nutritional standpoint, we organize and summarize all nanonutraceuticals for obesity therapeutics according to six categories of nutrients, that is, carbohydrates, lipids, proteins, vitamins, minerals, and phytochemicals (**Figure** **1**). In detail, we elucidate the characteristics, weight‐loss mechanisms, and bioavailability defects of each antiobese nutrient and its improved efficacy after nanomodification. Furthermore, we discuss the prospects, impediments, and solutions for the development of nanonutraceuticals in antiobesity applications. This review will not only assist researchers in gaining a rapid and comprehensive understanding of weight‐loss nanonutraceuticals and exploring them in‐depth but will also provide clinicians with a valuable reference for personalized nutraceutical treatments for obese patients.

## 2 Carbohydrates

Dietary fibers are carbohydrates with complex sugar chain structures, which are resistant to hydrolysis by digestive enzymes. Dietary fiber intake regulates intestinal integrity, gut microbiota, energy homeostasis, and body weight.^[^ ^21^, ^22^ ^]^ Two cross‐sectional studies and four prospective cohort studies have revealed a significant negative association between dietary fiber consumption and obesity.^[^ ^23^ ^]^ High dietary fiber intake reduces the risk of weight gain or obesity by about 30%. Currently, various nanomodified dietary fibers listed in **Table** **1** have been successfully applied in obesity intervention with positive outcomes.

**Table 1:** Advances in nanomodifications of carbohydrates, lipids, and proteins for weight loss.

Nutraceuticalsa)			Nanosizing	Size [nm]	Morphology	Solved issues	Antiobese effects	Mechanisms	Models	References
Carbohydrates	Cellulose	CNC	Acid hydrolysis	Width 4–7; Length 37–45	Nanocrystal	Poor interfacial adhesion; low melting point; water sensitive	Decreased BWG and food intake; decreased serum TG, TC, LDL‐C, and VLDL‐C	–	HFD rats	[30]
			–	–	Sphere	–	Decreased BWG and food intake; decreased serum TG, TC, LDL‐C, and non‐HDL‐C	Downregulation of hepatic bile acid metabolism and cholesterol synthesis‐related gene expression	Rats	[31]
		NFC	–	–	–	Insolubility in water; low viscosity (0.1–3%)	Decreased BWG and epididymal and subcutaneous fat accumulation	Gut microbiota balance	HFD mice	[33, 34]
		BC	High‐pressure homogenization	–	–	–	Decreased body weight and liver weight; decreased serum TC, TG, and LDL‐C	Improved insulin resistance; anti‐inflammation; antioxidation	HFD mice	[36]
	Chitin		Deacetylation and mechanical disintegration	–	–	–	Decreased serum TC, chylomicron, VLDL‐C, and phospholipid; decreased hepatic lipid accumulation	–	Rats	[42]
			Deacetylation and mechanical disintegration	Width 10–30; Length 150–500	Nanofiber/nanowhisker	–	Decreased BWG and ATW gain; decreased plasma and hepatic lipids	Lipid binding; emulsification	HFD mice	[40]
			Deacetylation and mechanical disintegration	–	–	–	Decreased body weight and liver weight; decreased serum TC, TG, and glucose and hepatic lipids	Lipid adsorption; gut microbiota reformation	NASH rats	[43]
	Chitosan		Ionic gelation and spray drying	400–700; 700–1000	Sphere	–	Decreased BWG, food intake, liver weight, and WAT weight; decreased serum TG, TC, and LDL‐C	–	HFD rats	[52]
			Ionic gelation and spray drying	500–800	Sphere	Poor water solubility; high viscosity	Decreased BWG; decreased serum TG, TC, and LDL‐C and plasma viscosity	–	HFD rats	[53]
			Ionic gelation and spray drying	600–1000	Sphere	Side effects	Decreased BWG; decreased serum TG, TC, and LDL‐C and plasma viscosity	–	HFD rats	[54]
Lipids	Medium‐chain fatty acids		Nanoliposome encapsulation	77.6 ± 4.3; 120.6 ± 10.1	–	Poor palatability; side effects	Decreased body weight and fat weight; decreased serum TC and TG	–	Mice	[65]
	Conjugated linoleic acid		Nanoemulsification	220.8 ± 7.0	Droplet	Poor water solubility; instability	Decreased body weight; decreased serum TG, TC, and LDL‐C and liver TG and TC	–	HFD rats; 3T3‐L1 cells	[70]
	Polyenyl‐phosphatidylcholine		–	–	–	–	High lipolytic effects	–	Mice; 3T3‐L1 cells	[79]
Proteins	Ganoderma lucidum protein		Nanoliposome encapsulation	149.8 ± 0.6	–	–	Decreased lipid accumulation	Down‐regulated expression of proteins related to lipid metabolism	3T3‐L1 cells	[85]
	Cytochrome c		PEGylated nanoparticle encapsulation	120.1 ± 15.4	–	Extracellular inactivation	Decreased BWG and fat mass; decreased serum leptin	Apoptosis of adipose EC	HFD mice; adipose EC	[89]
	Tamarind trypsin inhibitor		Chitosan/whey protein isolate encapsulation	109 ± 6.7	Sphere	Gastrointestinal instability	Decreased body weight	–	HGLI diet rats	[96]

a) Abbreviation: ATW, adipose tissue weight; BC, bacterial cellulose; BWG, body weight gain; CNC, cellulose nanocrystal; EC, endothelial cells; HDL‐C, high‐density lipoprotein cholesterol; HFD, high‐fat diet; HGLI, high glycemic index and high glycemic load; LDL‐C, low‐density lipoprotein cholesterol; NASH, nonalcoholic steatohepatitis; NFC, nanofibrillated cellulose; PEG, polyethylene glycol; TC, total cholesterol; TG, triglyceride; VLDL‐C, very low‐density lipoprotein cholesterol; WAT, white adipose tissue.

### 2.1 Cellulose

Cellulose is an insoluble dietary fiber, which is the most abundant renewable macromolecule in nature.^[^ ^24^ ^]^ As particle size decreases, cellulose exhibits an increase in specific surface area, water‐holding capacity, swelling capacity, mucoadhesive properties, and oil‐holding capacity.^[^ ^25^, ^26^ ^]^ Due to its unique physical and structural properties, nanocellulose may offer a promising approach for weight loss.^[^ ^27^ ^]^ There are three types of nanocellulose^[^ ^28^ ^]^ depending on their raw materials and processing methods: cellulose nanocrystal (CNC), nanofibrillated cellulose (NFC), and bacterial cellulose (BC).

#### 2.1.1 CNC

The main process for isolating CNC from cellulose fibers is based on acid hydrolysis. Paracrystalline regions of cellulose are preferentially hydrolyzed, whereas crystalline regions, which have higher resistance to acid attack, remain intact.^[^ ^29^ ^]^ CNC can be utilized as food fillers with the advantages of high hydrophilicity, high crystallinity, and high biocompatibility.

**Figure 2:**
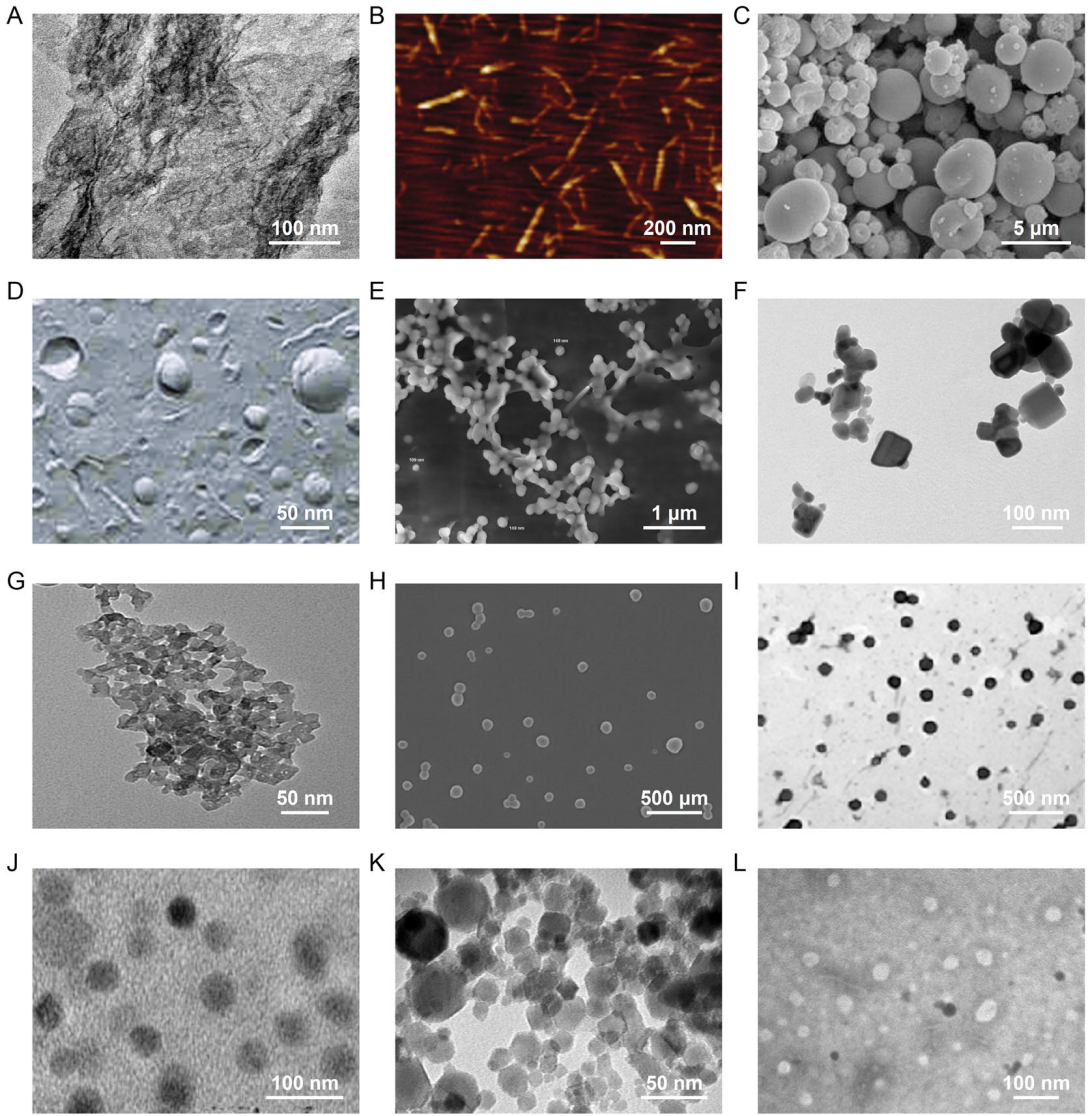
Morphology of various nanonutraceuticals. A) Representative transmission electron microscope (TEM) images of CNC. B) Representative atomic force microscope images of SDACNF. Representative scanning electron microscope (SEM) images of C) chitosan‐NP, D) N‐CLA, and E) TTI‐loaded CWNP. Representative TEM images of F) ZnO NP and G) nCrPic. H) Representative SEM images of resveratrol‐loaded PLGA NP. Representative TEM images of I) EGCG‐NP, J) quercetin‐loaded NP, K) NOC, and L) nanocelastrol. Source: (A) Reproduced with permission.^[^ ^30^ ^]^ Copyright 2016, ARC. (B) Reproduced with permission.^[^ ^40^ ^]^ 2018, Örebro University. (C) Reproduced with permission.^[^ ^54^ ^]^ Copyright 2011, Elsevier. (D) Reproduced with permission.^[^ ^70^ ^]^ Copyright 2013, Dove Medical Press. (E) Reproduced with permission.^[^ ^95^ ^]^ Copyright 2020, Elsevier. (F) Reproduced with permission.^[^ ^132^ ^]^ Copyright 2019, Elsevier. (G) Reproduced with permission.^[^ ^136^ ^]^ Copyright 2020, MDPI. (H) Reproduced with permission.^[^ ^151^ ^]^ Copyright 2018, The Royal Society. (I) Reproduced with permission.^[^ ^162^ ^]^ Copyright 2014, American Chemical Society. (J) Reproduced with permission.^[^ ^174^ ^]^ Copyright 2017, Elsevier. (K) Reproduced with permission.^[^ ^188^ ^]^ Copyright 2014, Dove Medical Press. (L) Reproduced with permission.^[^ ^194^ ^]^ Copyright 2019, Elsevier.

Abdelbaky et al.^[^ ^30^ ^]^ observed that the administration of CNC (**Figure** **2**A) isolated from red grape seeds to rats resulted in a significant reduction in their body weight gain (BWG) and food intake. Even rats that were fed an experimental diet containing only 2% CNC experienced a decrease in BWG, with mean values of −25.69 ± 2.20 g, compared with positive control rats, which had a BWG of 13.73 ± 1.91 g. The findings align with a study conducted by Lu et al.,^[^ ^31^ ^]^ which investigated the hypolipidemic effects of CNC derived from sweet potato residue in ovariectomized hyperlipidemic rats. Lu et al. also demonstrated that CNC can lower blood cholesterol levels, possibly due to its ability to impede endogenous hepatic cholesterol biosynthesis.

#### 2.1.2 NFC

There are many simple preparation processes for NFC, such as high‐pressure homogenization, high‐energy ball milling (mechanical chemistry), microfluidization, and ultra‐low temperature crushing.^[^ ^32^ ^]^ These mechanical methods are often combined with pretreatments, such as enzymatic hydrolysis, 2,2,6,6‐tetramethylpiperidine‐1‐oxyl radical (a piperidine nitroxide radical compound) oxidation, and other chemical modifications, to streamline the fibrillation process. The pretreatment can weaken the cellulose structure prior to mechanical processing into nanofibrils.

Both in vitro and in vivo experiments have evinced that the addition of NFC to a high‐fat diet (HFD) reduces fat digestion and absorption by decreasing triglyceride (TG) hydrolysis.^[^ ^27^ ^]^ According to scanning electron microscopy and molecular dynamics studies, this effect is mainly mediated by two mechanisms. The first mechanism involves the coalescence of fat droplets on NFC, which reduces the available surface area for lipase binding. The second mechanism involves the sequestration of bile salts, which disrupts protein interfacial displacement across the surface of lipid droplets and hinders the solubilization of lipid digestion products. In addition, Nagano et al.^[^ ^33^, ^34^ ^]^ observed that only 0.2% NFC consumption inhibited the increase in fat mass and body weight in HFD‐fed mice.

#### 2.1.3 BC

BC, a kind of highly crystalline cellulose without lignin and hemicellulose, is mainly produced by Acetobacter species via a biotechnological assembly process.^[^ ^35^ ^]^ BC supplementation can effectively reduce body, liver, and abdominal fat weights due to its multiple benefits.^[^ ^36^, ^37^ ^]^ As a fermentable source of colon microbiota, BC promotes the formation of short‐chain fatty acids, which aid in intestinal health, antioxidant defense, and lipid metabolism. In the liver, BC alleviates obesity‐related inflammation by decreasing tumor necrosis factor‐*α* (TNF‐*α*) and interleukin‐6 levels. In adipose tissues, BC modulates the secretion of adipocytokines and adipogenesis‐associated proteins. Additionally, Zhai et al.^[^ ^36^ ^]^ delved into the potential synergies of combining BC with other fibers. They found that the combination of BC and soluble fiber konjac glucomannan strongly enhanced antiobesity effects compared with BC or konjac glucomannan alone.

### 2.2 Chitin

Chitin, the second most abundant polysaccharide after cellulose, is the primary structural component found in the exoskeletons of crabs and shrimp, as well as in the cell walls of fungi and yeast. Chitin has been reported to reduce food utilization and adipose tissue accumulation in obese rats, thereby preventing BWG.^[^ ^38^ ^]^

Lipid hydrolysis was appreciably slower in the chitin nanocrystal‐stabilized emulsion than in the whey protein isolate‐ and sodium caseinate‐stabilized emulsions.^[^ ^39^ ^]^ Surface‐deacetylated chitin nanofibers (SDACNF) (Figure 2B), which have a highly uniform structure with a short‐axis diameter of 10–30 nm,^[^ ^40^, ^41^, ^42^ ^]^ offer a more promising alternative. With their submicron size and high surface‐to‐volume ratio, these nanofibers are more easily dispersed in water compared with chitosan and chitin powder. An in vitro study revealed that SDACNF exhibited higher binding affinity to total cholesterol (TC) and TG than deacetylated chitin. Moreover, the administration of SDACNF resulted in a significant decrease in BWG, liver weight, and adipose tissue weight (ATW) in mice.^[^ ^40^, ^43^ ^]^ The reduced ratio of ATW to body weight was also correlated with the dose of SDACNF. Histological examination further revealed that SDACNF were effective in decreasing hepatic lipid accumulation and preventing the development of steatohepatitis.

### 2.3 Chitosan

Chitosan is produced by deacetylating chitin in an alkaline medium or using chitin deacetylase. Chitosan is the only known natural linear cationic polysaccharide with biocompatible, biodegradable, and relatively nontoxic properties.^[^ ^44^ ^]^ It is suitable for various biomedical applications, such as obesity control and blood cholesterol reduction.^[^ ^45^, ^46^ ^]^ Chitosan lowers body weight by reducing fat absorption,^[^ ^45^ ^]^ suppressing adipocytokine secretion and fasting‐induced adipose factor expression,^[^ ^47^ ^]^ and improving intestinal microbiota.^[^ ^48^ ^]^ However, the optimal hypolipidemic effect of chitosan requires high doses, which may cause nausea and constipation.^[^ ^49^ ^]^ Hence, it is necessary to modify chitosan to improve its hypolipidemic activity.

Water‐soluble chitosan (WSC) has lower viscosity and higher reactivity compared with chitosan, which contributes to its lipid‐lowering efficacy.^[^ ^50^, ^51^ ^]^ Zhang et al.^[^ ^52^ ^]^ found that HFD rats treated with WSC‐microparticles (MP), chitosan‐MP, WSC‐NP, or chitosan‐NP gained significantly less body weight than rats fed only a normal diet or an HFD. The lowest BWG was recorded in the WSC‐MP‐treated rats. These four particles also reduced epididymal and perirenal white adipose tissue (WAT) weights. In addition, WSC‐ and chitosan‐NP (Figure 2C) not only effectively lowered plasma lipid levels and viscosity but also increased serum superoxide dismutase activities.^[^ ^52^, ^53^, ^54^ ^]^ In terms of safety, both WSC‐MP and WSC‐NP had relatively low acute oral toxicity.

## 3 Lipids

Most lipids found in daily diets are long‐chain fatty acids, which are preferentially stored as body fat.

In contrast, medium‐chain fatty acids (MCFA) and unsaturated lipids offer a significant dietary strategy for ameliorating obesity and its related metabolic dysbiosis via their capacity to regulate gut microbiota composition and epigenetics.^[^ ^55^, ^56^ ^]^ Hitherto, three nanomodified ingestible lipids have been reported for obesity management (Table 1).

### 3.1 MCFA

MCFA, composed of caprylic acid and capric acid, can be transported directly to the liver and oxidized rapidly without TG resynthesis, resulting in limited accumulation in adipose tissues.^[^ ^57^, ^58^ ^]^ Large amounts of MCFA are found in palm kernel and coconut. Research has demonstrated that the consumption of MCFA may be beneficial in the dietary management of weight loss.^[^ ^59^, ^60^, ^61^, ^62^, ^63^ ^]^ There are two primary reasons for this benefit: heightened satiety and enhanced fat oxidation in brown adipose tissue. Nevertheless, human subjects are unable to consume high‐MCFA diets on a long‐term basis due to adverse gastrointestinal symptoms and a lack of palatability.^[^ ^64^ ^]^

To overcome the drawbacks of MCFA, Liu et al.^[^ ^65^ ^]^ encapsulated MCFA into nanoliposomes (NL) and investigated the body fat‐reducing properties of MCFA NL in mice over both short‐term (2 weeks) and long‐term (6 weeks) periods. No abnormal clinical signs and gross pathological abnormalities were observed in tissues and organs after short‐term administration of MCFA NL at a dose of 1000 mg kg^−1^. In the long‐term study, mice in the MCFA NL group exhibited normal food intake in contrast to the MCFA group, which indicates that MCFA NL can overcome the poor palatability of MCFA. In that case, MCFA NL suppressed body fat accumulation and reduced serum TC and TG levels in the same way as MCFA.

### 3.2 Conjugated Linoleic Acid (CLA)

CLA is a group of positional and geometric isomers of linoleic acid characterized by the presence of conjugated dienes.^[^ ^66^ ^]^ The major dietary source of CLA for human is ruminant meats and dairy products. Dietary CLA supplementation can markedly reduce body fat mass and improve lipid metabolism.^[^ ^67^, ^68^ ^]^ CLA supplementation reduces adiposity by suppressing appetite, increasing energy expenditure and lipolysis, reducing adipogenesis, and triggering adipocyte apoptosis.^[^ ^69^ ^]^ However, CLA exhibits poor water solubility and high oxidation sensitivity.

Nanoemulsified water‐soluble conjugated linoleic acid (N‐CLA) (Figure 2D) was developed to solve these problems.^[^ ^70^ ^]^ In vitro research, N‐CLA had a stronger lipolytic effect on mature adipocytes compared with CLA. Leptin secretion was significantly enhanced by N‐CLA to a similar extent as orlistat, an antiobesity medication approved by the Food and Drug Administration (FDA). In obese rats fed an HFD, N‐CLA was more efficient than CLA in reducing body weight and serum levels of TC, TG, and low‐density lipoprotein cholesterol (LDL‐C). Although N‐CLA with high bioavailability has greater antiobesity effects in comparison with CLA, further research is needed to reveal its mechanism for combating obesity and potential side effects like hyperinsulinemia.^[^ ^71^ ^]^

### 3.3 Polyenyl‐Phosphatidylcholine (PPC)

PPC, a purified polyunsaturated phosphatidylcholine extract from soybeans, has been used for indications such as regulating blood lipid, preventing fat embolism, and even treating fatty liver.^[^ ^72^, ^73^ ^]^ Subcutaneously injected PPC plus deoxycholate has been proven to reduce adipose tissue volume in several clinical studies.^[^ ^74^, ^75^, ^76^ ^]^ However, in vitro experiments found that this compound lyses and kills not only adipocytes but also normal cells such as vascular smooth muscle cells, skeletal muscle cells, and renal epithelial cells.^[^ ^77^, ^78^ ^]^

NWL‐10 is a novel mixture prepared with 50 mg mL^−1^ PPC in nanoparticle size (<40 nm), 20 mg mL^−1^ glycyrrhizinate, and 180 mg mL^−1^ maltose for lipolytic action on adipose tissue.^[^ ^79^ ^]^ NWL‐10 showed little cytotoxicity but high lipolytic effects, which increased with ascending concentrations in human adipose tissue‐derived stem cells and mouse 3T3‐L1 cells. Moreover, neither bioluminescence monitoring nor histological results showed any inflammatory effects caused by NWL‐10 in mice.

## 4 Proteins

Large randomized trials and meta‐analyses have demonstrated that high‐protein diets result in more significant weight loss during the initial rapid weight‐loss phase (3–6 months) compared with regular low‐fat or high‐carbohydrate diets. This may be due to a more negative energy balance as a result of enhanced diet‐induced thermogenesis, better preservation of fat‐free mass, and improved satiety with higher dietary protein intake.^[^ ^80^ ^]^ In addition to protein quantity, protein source, and specific peptides and amino acids can also positively influence the variables and complications of obesity.^[^ ^81^, ^82^ ^]^ Although proteins and bioactive protein hydrolysates may lose their activity partially or completely before reaching target cells or organs through oral administration, the use of nanodelivery systems can improve their stability and bioavailability in the human physiological environment and during commercial processing. Three nanoencapsulated special proteins have been utilized with satisfactory results in antiobesity applications (Table 1).

### 4.1 Ganoderma Lucidum (GL) Protein

GL is a medicinal fungus that contains polysaccharides, triterpenoids, and proteins as its main pharmacological ingredients. With the application of new separation and purification technologies, more bioactive peptides and proteins from GL have been documented. GL protease hydrolysate has been demonstrated to improve lipid metabolism disorders and regulate the composition of intestinal microbes in rats fed an HFD.^[^ ^83^ ^]^ Liposome encapsulation is appropriate for delivering proteins and peptides since their molecules possess both polar and nonpolar areas similar to liposome characteristics.^[^ ^84^ ^]^ One study^[^ ^85^ ^]^ found that GL protein hydrolysate‐loaded NL made from cholesterol and lecithin were highly stable in the gastric environment and pancreatin. These NL induced cellular lipolysis without impacting cell viability. Glycerol release was 1.63 ± 0.25 times higher than that of the control. Moreover, label‐free proteomics revealed that fatty acid synthase, a crucial player in lipogenesis, was suppressed more than fivefold in the loaded NL group. Thus, nanoliposomal GL protein can be regarded as a reliable inhibitor of lipid accumulation.

### 4.2 Cytochrome *c* (Cyt *c*)

By inducing endothelial cell apoptosis and inhibiting neovascularization in white fat, it is possible to prevent the formation of new adipocytes and adipocyte hypertrophy.^[^ ^86^ ^]^ Cyt *c* is a physiologically adaptable mitochondrial protein that plays a significant role in mediating apoptosis. However, Cyt *c* is highly biodegradable with a biological half‐life of 4 min. It is adsorbed easily by blood proteins and subsequently interacts with phagocytes, which leads to its rapid clearance from the body.^[^ ^87^ ^]^ To effectively exert the antiobesity function of Cyt *c*, it is necessary to specifically deliver exogenous Cyt *c* into the cytoplasm of endothelial cells in adipose tissues.

A prohibitin (a white fat vessel‐specific receptor)‐targeted nanoparticle (PTNP) system has been reported to actively accumulate in vascular endothelial cells of adipose tissues in mice, while blood vessels of other organs like spleen, brain, heart, and liver are exempted.^[^ ^88^, ^89^ ^]^ Benefiting from the intactness of PTNP within circulation, exogenous Cyt *c* can be transported into the cytoplasm of vascular endothelial cells in adipose tissues in the form of an aqueous internal core and then activate caspase cascade at minute dosages to induce apoptosis. Hossen et al.^[^ ^89^ ^]^ observed that Cyt *c*‐loaded PTNP administration reduced the percentage of BWG in HFD‐fed mice in a dose‐dependent manner without detectable hepatoxicity. The size of subcutaneous and epididymal fats was also reduced. Apparently, Cyt *c* exhibited excellent antiobesity efficacy with the assistance of the PTNP system.

### 4.3 Tamarind Trypsin Inhibitor (TTI)

Tamarind seeds are a source of trypsin inhibitors. TTI promotes satiety by increasing cholecystokinin, reducing plasma leptin, and ameliorating inflammation.^[^ ^90^, ^91^, ^92^, ^93^ ^]^ It did not cause any signs or symptoms of general toxicity or damage to the intestine, stomach, liver, and pancreas in obese rats at the bioactive dose of 25 mg kg^−1^. However, it is noteworthy that the effect of TTI on satiety did not lead to weight loss when TTI was administered to obese animals.^[^ ^92^ ^]^

Chitosan‐whey protein nanoparticles (CWNP) with good chemical stability have been proved to be an effective oral delivery system for TTI, which can be released under the conditions of gastric and intestinal digestion.^[^ ^94^ ^]^ According to Costa et al.,^[^ ^95^ ^]^ no instances of toxicity related to TTI‐loaded CWNP (Figure 2E) have been reported in both in vitro and in vivo studies. In a preclinical study,^[^ ^96^ ^]^ TTI‐loaded CWNP significantly reduced body weight in Wistar rats without enhancing anti‐inflammatory effects. CWNP could potentiate satiety in combination with TTI. The remarkable weight loss was probably caused by a negative mean variation in dietary intake.

## 5 Vitamins

Vitamins are organic compounds essential for maintaining health and supporting bodily functions. Previous studies have indicated that the consumption of multivitamins is inversely correlated with body weight, body mass index (BMI), and body composition, which is attributed to their anti‐inflammatory, antioxidant, antiadipogenic, and other biological effects.^[^ ^97^, ^98^ ^]^ In particular, vitamin A and vitamin D have been nanomodified to combat obesity (**Table** **2**).

**Table 2:** Advances in nanomodifications of vitamins and minerals for weight loss.

Nutraceuticalsa)		Nanosizing	Size (nm)	Morphology	Solved issues	Antiobese effects	Mechanisms	Models	References
Vitamins	Vitamin A	Scallop PL encapsulation	121.0 (38.7–296.2)	–	–	Decreased body weight and total WAT weight	Upregulation of fat *Ucp‐1* expression	KK‐A(y) mice	[107]
	Vitamin D	Nanoemulsification	–	Droplet	Poor water solubility; chemical degradation	Decreased serum TC, TG, and TNF‐*α*; increased serum HDL‐C and IL‐10; improved liver steatosis	Upregulation of hepatic *Vdr*, *Nrf2*, and *Cpt1α* expression	HFD rats	[114]
		Nanocapsulation	126.1 ± 10.8	–	–	Decreased WHR; decreased serum TG, insulin, and inflammatory cells; increased serum HDL‐C	–	Human with obesity	[115, 116]
Minerals	Zinc	–	30 ± 10; 90 ± 10	–	Low intestine absorption	Decreased body weight, fat index, and adipocytes; decreased serum TC, TG, LDL‐C, glucose, IL‐1*β*, and LPS‐binding protein	–	HFD mice	[131]
		–	38.5 ± 2.8	–	–	Decreased lipid accumulation in hepatocytes; decreased BWG, fat weight, and hepatic fat accumulation	Hepatic SIRT1‐LKB1‐AMPK activation	HFD mice; HepG2 cells	[132]
	Chromium	Grounding and sieving	49.7 ± 12.4	–	Low intestine absorption	Decreased subcutaneous fat thickness; improved insulin resistance	Upregulation of *ADIPOQ* expression in adipose tissues	Pigs	[136, 137]

a) Abbreviation: AMPK, adenosine monophosphate‐activated protein kinase; BWG, body weight gain; HDL‐C, high‐density lipoprotein cholesterol; HFD, high‐fat diet; IL‐10, interleukin‐10; IL‐1*β*, interleukin‐1*β*; LDL‐C, low‐density lipoprotein cholesterol; LKB1, liver kinase B1; LPS, lipopolysaccharide; PL, phospholipid; SIRT1, silent mating‐type information regulation 2 homolog 1; TC, total cholesterol; TG, triglyceride; TNF‐*α*, tumor necrosis factor‐*α*; WAT, white adipose tissue; WHR, waist‐to‐hip ratio.

### 5.1 Vitamin A

Vitamin A refers to a family of hydrophobic compounds including retinoids (primarily retinol and retinyl esters) and carotenoids, which are essential for maintaining growth and development, male and female reproduction, barrier integrity, immunity, and vision.^[^ ^99^, ^100^, ^101^ ^]^ Vitamin A deficiency has been found in obese individuals.^[^ ^102^ ^]^ Numerous experiments have demonstrated that retinoids and carotenoids are important regulators in adipose tissue development.^[^ ^103^ ^]^ They can inhibit adipocyte differentiation and oxidative stress to exert antiobesity actions. According to a meta‐analysis conducted by Yao et al.,^[^ ^104^ ^]^ carotenoid supplementation was significantly associated with weight reduction, BMI decrease, and waist circumference loss.

Fucoxanthin is a characteristic carotenoid present in brown seaweeds, such as *Undaria pinnatifida* and *Laminaria japonica*. In recent years, its antiobesity and antidiabetic effects have been frequently reported due to its unique therapeutic mechanism.^[^ ^105^, ^106^ ^]^ To further enhance the antiobesity potential of fucoxanthin, Okada et al.^[^ ^107^ ^]^ nanoencapsulated fucoxanthin with scallop phospholipids rich in n‐3 polyunsaturated fatty acids that affect lipid metabolism. The encapsulated fucoxanthin resulted in a remarkable reduction in body weight and total white fat mass in male KK‐A^y^ mice compared with the control. This effect was probably attributed to the upregulation of *Ucp‐1* (a gene related to adipose browning) expression in WAT.

### 5.2 Vitamin D

As a typical fat‐soluble vitamin, vitamin D is essential for the normal functioning of various physiological processes. Traditionally, vitamin D has been primarily linked to the regulation of calcium and phosphorus metabolism.^[^ ^108^ ^]^ Despite its discovery a century ago, the multifaceted benefits of vitamin D have only been elucidated in recent decades.^[^ ^109^ ^]^ These benefits encompass significant antioxidative,^[^ ^110^ ^]^ anti‐inflammatory,^[^ ^111^ ^]^ and antifibrotic^[^ ^112^ ^]^ effects. Furthermore, ongoing research is actively investigating the potential of vitamin D supplementation in mitigating metabolic disorders induced by overweight and obesity. A study conducted by Mikuska et al.^[^ ^113^ ^]^ indicated that vitamin D intake may be an effective strategy to reduce body weight and abdominal adiposity.

An inescapable fact is that vitamin D has poor water solubility and is excreted slowly in urine, which may result in toxicity in the event of excessive accumulation.^[^ ^109^ ^]^ Therefore, enhancing the solubility of vitamin D is crucial to maximize its efficacy and minimize side effects. El‐Sherbiny et al.^[^ ^114^ ^]^ found that vitamin D nanoemulsion was more effective than conventional oral vitamin D as a hepatoprotective formulation in HFD‐fed rats, due to its improved absorption and bioavailability. Moreover, a parallel double‐blind randomized clinical trial^[^ ^115^ ^]^ revealed a correlation between the intake of nanoencapsulated vitamin D_3_ from dairy products and improvements in certain obesity‐related parameters such as anthropometric indexes, glucose homeostasis, and lipid profiles. These dairy products also decreased inflammation levels in individuals with abdominal obesity.^[^ ^116^ ^]^

## 6 Minerals

Like vitamins, the human body's requirement for minerals is relatively low. However, as inorganic elements, minerals are also vital nutrients. Accumulating evidence^[^ ^117^, ^118^ ^]^ has revealed that mineral deficiency is a risk factor for obesity and related complications. Conversely, targeted mineral supplementation has been shown to be beneficial in reducing obesity or BMI, especially in nanosized forms (Table 2).

### 6.1 Zinc

Zinc, an essential mineral, controls the catalytic activity of more than 300 enzymes in vivo. It is involved in numerous biological functions and plays a pivotal role in immune system,^[^ ^119^ ^]^ wound healing,^[^ ^120^, ^121^ ^]^ learning and memory,^[^ ^122^, ^123^ ^]^ protein synthesis,^[^ ^124^ ^]^ DNA synthesis,^[^ ^125^, ^126^ ^]^ and cell proliferation.^[^ ^126^ ^]^ Epidemiological studies have revealed that obese and overweight individuals have low serum zinc concentrations.^[^ ^127^, ^128^ ^]^ Zinc supplementation is beneficial to improve their BMI, body weight, serum TG levels, and even obesity‐related inflammation.^[^ ^129^, ^130^ ^]^

Zinc oxide (ZnO) is a common food additive used to fortify foods with zinc. Although ZnO is insoluble in water, its absorption and bioavailability in the gastrointestinal tract can be improved by making it into NP. Liu et al.^[^ ^131^ ^]^ compared the effects of bulk ZnO and ZnO NP (30 or 90 nm) on obesity phenotypes, including glucolipid metabolism and inflammation profiles, in HFD‐induced obese mice. Their results demonstrated that both bulk and nanosized ZnO could attenuate obesity phenotypes, but nanosized ZnO was more effective in reducing fat index. Moreover, another study found that ZnO NP (Figure 2F) had the ability to significantly alleviate HFD‐induced hepatic steatosis through the adenosine monophosphate‐activated protein kinase (AMPK) signaling axis in its activated state.^[^ ^132^ ^]^

### 6.2 Chromium

Chromium (III), or trivalent chromium, is an essential trace element that is ubiquitous in foods at low concentrations and available as a dietary supplement. Chromium plays a role in the regulation of carbohydrate, lipid, and protein metabolism by enhancing insulin efficacy.^[^ ^133^, ^134^ ^]^ Oral chromium supplementation may be associated with improvements in body weight and body composition, especially fat percentage, in individuals with overweight/obesity. A meta‐analysis reviewed the effect of chromium supplementation on anthropometric indices.^[^ ^135^ ^]^ This work included 1316 participants across 21 trials with durations ranging from 9 to 24 weeks. Those who consumed 200–1000 μg of chromium per day experienced greater weight loss and reductions in BMI and body fat percentage compared with placebo.

However, the oral bioavailability of common chromium supplements has been reported to be suboptimal, which affects their practical applications. In this regard, some studies have pointed out that reducing the particle size of chromium can alleviate this problem and enhance its bioavailability. Hung et al.^[^ ^136^, ^137^ ^]^ prepared nano‐chromium picolinate (nCrPic) (Figure 2G) particles with an average size of 49.7 ± 12.37 nm and added them to pig feed to compare their effects with those of ordinary chromium picolinate (CrPic). The results showed that dietary nCrPic reduced both subcutaneous fat thickness and cadaver weight in pigs compared with CrPic treatment. Moreover, dietary nCrPic was effective in attenuating insulin resistance. Further research indicated that these effects were achieved by decreasing suppressor of cytokine signaling 3 and increasing uncoupling protein 3 and interleukin‐15 in skeletal muscle, while increasing adiponectin in subcutaneous adipose tissue.

## 7 Phytochemicals

Phytochemicals, albeit not nutrients in the traditional sense, exhibit diverse health benefits for humans. Oral intake of specific phytochemicals has been reported to prevent and treat obesity and related metabolic diseases via multifarious pathways, such as altering ceramide accumulation, promoting fat browning, and inhibiting adipogenesis.^[^ ^138^, ^139^, ^140^ ^]^ However, their oral bioavailability and bioefficacy tend to be poor. To date, a variety of phytochemicals have undergone nanomodification to enhance weight‐loss effects (**Table** **3**).

**Table 3:** Advances in nanomodifications of phytochemicals for weight loss.

Nutraceuticalsa)	Nanosizing	Size [nm]	Morphology	Solved issues	Antiobese effects	Mechanisms	Models	References
Lutein	Nanoemulsification	254.2	–	Low intestine absorption	Decreased hepatic steatosis and cholesterol accumulation; decreased hepatic OxLDL and IL‐1*β*	–	HCD pigs	[145]
Resveratrol	PLGA nanoparticle encapsulation	176.1	Sphere	Poor aqueous solubility; instability; intestinal metabolism	Decreased hepatocyte lipid accumulation and proliferation; increased lipolysis	–	HepG2 cells	[151]
Epigallocatechin gallate	CS‐PPA nanoparticle encapsulation	100.6 ± 4.2	Sphere	Instability	Decreased arterial lipid deposition; decreased serum TG, TC, and LDL‐C	–	HFD rabbits	[162]
Anthocyanins	Chitosan nanoparticle encapsulation	–	–	Instability	Decreased body weight; decreased serum TC, TG, LDL‐C, and VLDL‐C; increased HDL‐C	Decreased expression of hepatic enzymes related to lipid metabolism	HFAD rats	[168]
Quercetin	Chitosan/alginate nanoparticle encapsulation	Minimum ≈91.6	Sphere	Poor aqueous solubility and permeability; instability	Decreased serum TG, TC, and glucose	–	Diabetic rats	[174]
Oleanolic acid	Chitosan nanoparticle encapsulation	–	–	Low solubility in both water and oil	Decreased body weight; decreased serum TC, TG, insulin, and ISI; decreased hepatic lipid accumulation	–	HFFD rats	[180]
Capsaicin	Self‐assembly	159.1 ± 1.6	Sphere	Poor aqueous solubility	Decreased TC, TG, LDL‐C, and TBA; decreased hepatic lipids; increased HDL‐C	–	HFD rats	[187]
	Nanoemulsification	20–50	–	Poor solubility	Decreased body weight and adipose tissue mass; decreased plasma TG	Expression changes of adipogenesis, *β*‐oxidation, and thermogenesis‐related genes in WAT; AMPK activation and GPDH inhibition in WAT	HFD rats	[188]
	Nanoemulsification	167.9 ± 0.3	Droplet	–	Decreased BWG, adipose tissue mass, and adipocyte size; decreased serum TC, TG, and LDL‐C; improved hepatic steatosis	–	HFD rats	[189]
Celastrol	PEG‐PCL nanomicelle encapsulation	50–70	Sphere	Poor aqueous solubility and high‐dose toxicity	Decreased body weight and fat mass; decreased lipid accumulation and adipocyte hypertrophy in liver and adipose tissues; improved insulin insensitivity	–	HFD mice	[194]

a) Abbreviation: AMPK, adenosine monophosphate‐activated protein kinase; BWG, body weight gain; CS‐PPA, chitosan and polyaspartic acid; GPDH, glycerol‐3‐phosphate dehydrogenase; HCD, hypercholesterolemic diet; HDL‐C, high‐density lipoprotein cholesterol; HFAD, high fat‐alcohol diet; HFD, high‐fat diet; HFFD, high fat and fructose diet; IL‐1*β*, interleukin‐1*β*; ISI, insulin sensitivity index; LDL‐C, low‐density lipoprotein cholesterol; OxLDL, oxidized low‐density lipoprotein; PEG‐PCL, poly(ethylene glycol)‐poly(ε‐caprolactone) copolymers; PLGA, poly (lactic*‐co*‐glycolic acid); TBA, total bile acid; TC, total cholesterol; TG, triglyceride; VLDL‐C, very low‐density lipoprotein cholesterol; WAT, white adipose tissue.

### 7.1 Lutein

Lutein, a type of carotenoid compound, is particularly abundant in light‐exposed plants (broccoli, spinach, and peas) and other foods such as eggs. Lutein in food substances possesses significant functions in human health, especially eye and cerebral health, because of its antioxidant properties. Compelling evidence in primates has also demonstrated the positive role of lutein in preventing age‐related macular degeneration and cataracts.^[^ ^141^ ^]^ In terms of antiobesity, lutein has been reported to impede adipocyte differentiation in vitro and ameliorate obesity, fatty liver, and glucose intolerance in mice fed an HFD.^[^ ^142^, ^143^ ^]^

However, lutein bioavailability is often poor, with only a tiny percentage being absorbed and utilized in the alimentary tract.^[^ ^144^ ^]^ Facing this issue, Murillo et al.^[^ ^145^ ^]^ fabricated an oil‐in‐water nanoemulsion (particle size: 254.2 nm and polydispersity index: 0.29) for the oral delivery of lutein. They compared the bioavailability of powdered lutein (PL) and lutein nanoemulsion, as well as their effects on metabolic variables in plasma, liver, and fat in a guinea pig model of hepatic steatosis. As anticipated, lutein nanoemulsion had higher bioavailability than PL. Moreover, lutein nanoemulsion exerted a protective action against cholesterol‐induced liver damage by reducing oxidation, lipid accumulation, and the release of proinflammatory factors (e.g., interleukin‐1beta).

### 7.2 Resveratrol

Resveratrol, a polyphenolic compound primarily found in peanut sprouts, grapes, and peanuts,^[^ ^146^ ^]^ was initially identified as a natural phytotoxin for its ability to protect plants against bacterial and fungal attacks. However, it has gained significant attention within the scientific community in recent years for its diverse biological effects, including antioxidant, anticancer, antiaging, antidiabetic, cardioprotective, and neuroprotective properties.^[^ ^147^ ^]^ Notably, resveratrol also shows substantial benefits for energy metabolism and metabolic‐related diseases such as obesity. Li et al.^[^ ^148^ ^]^ revealed that resveratrol can induce WAT browning and reduce fat accumulation by modulating the nicotinamide adenine dinucleotide‐dependent deacetylase silent information regulator 1, thereby ameliorating hyperglycemia and hyperlipidemia in mice. However, resveratrol exhibits very low bioavailability, with only a small fraction (≈1%) of orally ingested resveratrol being utilized by the body in its bioactive form.^[^ ^149^ ^]^

Nanocarrier technology is extensively utilized in the oral delivery of polyphenolic compounds and offers a promising solution to overcome the low bioavailability of resveratrol. Zu et al.^[^ ^150^ ^]^ successfully formulated resveratrol encapsulated lipid nanocarriers and resveratrol encapsulated liposomes, which not only enhance the water solubility and chemical stability of resveratrol but also further augment its ability to induce adipose browning. Poly lactic*‐co*‐glycolic acid (PLGA) NP have also been demonstrated to enhance the stability, solubility, and bioactivity of resveratrol. Wan et al.^[^ ^151^ ^]^ prepared resveratrol‐loaded PLGA NP (Figure 2H) with high encapsulation efficiency (97.25%) and drug loading (14.9%) using an oil/water emulsion technology. These NP exhibited remarkable stability under extremely acidic (pH 1.2) and high temperature (48 °C) conditions, as well as potent inhibition of lipid accumulation in steatotic HepG2 cells. Similarly, the incorporation of plant‐derived starch to encapsulate resveratrol could enhance its activities against diabetes and obesity.^[^ ^152^ ^]^ Furthermore, this resveratrol nanoencapsulated by starch did not compromise its biological benefits even when added to wheat flour to produce palatable snacks. Such snacks had higher resveratrol retention (43–53%) and still showcased superior antioxidant, antidiabetic, and antiobesity activities in comparison to control snacks with free resveratrol added.^[^ ^153^ ^]^

### 7.3 Epigallocatechin Gallate (EGCG)

EGCG, the main bioactive ingredient in green tea, is a flavone‐3‐ol polyphenolic substance with eight hydroxyl groups that is important for its bioactivities such as antiobesity, cardioprotective, and neuroprotective effects.^[^ ^154^, ^155^ ^]^ The weight‐loss effect of EGCG involves decreasing energy absorption and enhancing fat oxidation through the regulation of lipid metabolism‐related gene expression.^[^ ^156^, ^157^ ^]^ This effect presents a favorable EGCG dose dependence. A high dose of EGCG was required to achieve weight loss in clinical trials.^[^ ^158^ ^]^ However, this therapeutic dose may not be far from the threshold of toxicity. The bioavailability of EGCG after oral administration is about 0.1% in both humans and animals, which can be attributed to its low stability, poor cellular uptake, premature degradation, and active efflux.^[^ ^159^, ^160^, ^161^ ^]^

Accordingly, Hong et al.^[^ ^162^ ^]^ prepared EGCG‐NP (Figure 2I) by self‐assembly of chitosan and polyaspartic acid to overcome the obstacles of EGCG bioavailability. The release of EGCG from EGCG‐NP under different pH conditions showed that these NP were stable in the stomach and released rapidly in the intestine, thus potentially improving the intestinal absorption of EGCG. Daily administration of EGCG‐NP containing only 100 mg EGCG lowered serum TG, TC, and LDL‐C levels in rabbits by 52%, 55%, and 65%, respectively. This finding highlighted the remarkable efficacy of EGCG‐NP in reducing serum lipid levels, which entirely corresponded to the decrease in lipid deposition. Based on this work, it can be inferred that the antiatherosclerotic effect of EGCG‐NP is comparable to that of simvastatin, a first‐line medication widely prescribed for atherosclerosis treatment.

### 7.4 Anthocyanins

Anthocyanins, which belong to the group of polyphenolic compounds, are widely distributed and function as water‐soluble pigments in nature. These compounds are abundant in colored flowers or fruits such as cherries and pomegranates. Notably, their distinctive property lies in their ability to display varying tones of blue, red, and purple based on pH value.^[^ ^163^ ^]^ These vibrant colors play a crucial role in attracting animals for seed dispersal and pollination, as well as shielding plants from ultraviolet radiation‐induced damage through light absorption.^[^ ^164^ ^]^ Apart from replacing artificial food colorants within the food industry realm, anthocyanins possess eminent antioxidant, anti‐inflammatory, and anticancer activities.^[^ ^165^ ^]^ In recent years, there has been growing attention towards exploring the potential effects of anthocyanins on weight loss. Song et al.^[^ ^166^ ^]^ have demonstrated that anthocyanins can modulate gut microbiota to alleviate HFD‐induced obesity and hepatic steatosis. However, they are susceptible to degradation under conditions such as high temperature, nonoptimal pH, and oxygen exposure. Such poor stability may reduce their effectiveness in biological actions.

Nanoencapsulation is considered the most suitable approach to address this issue, as it enhances the molecular stability, bioavailability, and controlled release of anthocyanins within the gastrointestinal tract.^[^ ^167^ ^]^ For example, Sreerekha et al.^[^ ^168^ ^]^ prepared anthocyanin‐loaded chitosan nanoparticles (ACNP) using chitosan as a reducing and stabilizing agent and tripolyphosphate as a cross‐linking agent. The results showed that supplementation with ACNP led to more significant weight loss in rats compared with anthocyanin alone under the same dietary regimen (i.e., HFD and daily alcohol consumption). Moreover, ACNP‐supplemented rats had lower levels of serum TG, LDL‐C, and very low‐density lipoprotein cholesterol (VLDL‐C). These beneficial changes might result from the ACNP‐mediated downregulation of 3‐hydroxy‐3‐methylglutaryl coenzyme A reductase, an enzyme responsible for cholesterol biosynthesis.

### 7.5 Quercetin

Quercetin, a natural bioactive compound based on the flavone structure C6 (ring A)‐C3 (ring C)‐C6 (ring B), is widely present in fruits and vegetables like onions.^[^ ^169^, ^170^ ^]^ As a special subclass of flavonoids, quercetin has antioxidant, antiviral, anti‐inflammatory, and antiobesity properties. Seo et al.^[^ ^171^ ^]^ have demonstrated that quercetin can reduce body weight in a dose‐dependent manner (notably, a 40% reduction at a dose of 100 mg kg^−1^ for 10 weeks) and inhibit lipogenesis in HFD‐induced obese mice. A 12‐week, randomized, double‐blind, placebo‐controlled clinical trial^[^ ^172^ ^]^ also revealed that 100 mg day^−1^ of quercetin significantly reduced body weight and body fat percentage. These effects were not observed in the placebo group. The antiobesity effect of quercetin may be related to the activation of AMPK, the inhibition of c‐Jun N‐terminal kinase, and the regulation of mitogen‐activated protein kinase.^[^ ^173^ ^]^

However, quercetin's application is hampered by its poor aqueous solubility, chemical instability (especially in aqueous alkaline medium), and poor permeability. To circumvent these problems, Mukhopadhyay et al.^[^ ^174^ ^]^ encapsulated quercetin into pH‐sensitive core–shell NP composed of alginate and succinyl chitosan through ionic cross‐linking, resulting in quercetin‐loaded NP (Figure 2J) with a minimum particle size of about 91.58 nm. The results of animal experiments showed that mice treated orally with quercetin‐loaded NP exhibited a more significant decrease in cholesterol and TG levels in comparison with mice treated with free quercetin. This enhanced hypolipidemic effect could be attributed to the ability of core–shell NP to retard the release of quercetin in the stomach and ensure a sustained release in the intestine.

### 7.6 Oleanolic Acid (OA)

OA is a natural triterpene commonly present in fruits and vegetables like olive leaves, grapes, and pomegranates. Previous studies indicate that OA has various pharmacological and biochemical effects, including anti‐inflammatory, antioxidant, and hypoglycemic effects.^[^ ^175^, ^176^ ^]^ Additionally, De Melo et al.^[^ ^177^ ^]^ observed that OA could ameliorate visceral obesity and glucose tolerance in HFD‐induced obese mice by regulating carbohydrate and fat metabolism. This finding about OA's antiobesity aligns with the research conducted by Li et al.^[^ ^178^ ^]^ Intriguingly, OA was found to upregulate CD36 mRNA expression in taste bud cells, modulate intracellular CD36 receptor‐related events, and improve gustatory perception of lipids, thereby exerting antiobesity effects.^[^ ^179^ ^]^

However, the bioavailability of OA is limited by its relatively low solubility in both oil and water. To tackle this issue, Wang et al.^[^ ^180^ ^]^ devised a nanoformulation of OA via a self‐assembly approach and then assessed the effectiveness of nano‐OA in treating insulin resistance and metabolic disorders in high fat and fructose diet (HFFD)‐fed rats. The results showed that HFFD‐fed rats treated with nano‐OA experienced decreases in body weight, serum insulin, insulin sensitivity index, and serum TC and TG. Moreover, the beneficial effects of nano‐OA were better than those of OA and rosiglitazone. In brief, this work substantiated that nano‐OA could effectively ameliorate HFFD‐induced metabolic dysfunctions by enhancing bioavailability.

### 7.7 Capsaicin

Widely known for their spicy flavor, chili peppers are one of the most commonly used spices in the world. Capsaicin (*trans*‐8‐methyl‐N‐vanillyl‐6‐nonenamide), a major alkaloid found in chili peppers, acts as a natural defense molecule to safeguard immature pepper seeds from insect infestation. Numerous reports indicate that capsaicin is beneficial for pain relief, inflammation reduction, cancer prevention, and cardiovascular health.^[^ ^181^, ^182^, ^183^, ^184^ ^]^ It is noteworthy that both in vitro and in vivo studies have demonstrated capsaicin's capability to induce WAT browning, which results in a reduction of fat content. This physiological action is postulated to occur through the activation of signaling pathways that involve *β*3‐adrenergic receptor and peroxisome proliferator‐activated receptor‐*γ*.^[^ ^185^ ^]^ Nonetheless, capsaicin's practical application in weight loss has encountered various obstacles for its limited water solubility. Moreover, capsaicin causes stomach discomfort when ingested and cutaneous and ocular irritation when applied topically.^[^ ^186^ ^]^

Some studies in animal models have pointed out that nanotechnology can address the issues of inadequate absorption and irritant nature of capsaicin. Self‐assembled capsaicin prodrug NP exhibited improved aqueous solubility, along with enhanced lipid‐lowering activity and reduced mucosal irritation.^[^ ^187^ ^]^ Kim et al.^[^ ^188^ ^]^ formulated a premixture by combining oleoresin capsicum (OC) with an equal amount of Tween 80 to produce nanoemulsion OC (NOC) (Figure 2K). Compared with OC‐treated obese rats, NOC‐treated obese rats showed significant weight loss (10.2%) and lower plasma TC concentration, although there were no appreciable alternations in energy intake and energy efficiency ratio. A study conducted by Lu et al.^[^ ^189^ ^]^ also corroborated the above findings. They evaluated the antiobesity effects of capsaicin‐loaded nanoemulsions (C‐NE) on male rats fed an HFD. The results showed that C‐NE could alleviate capsaicin‐caused irritation in gastric tissue and HFD‐induced hepatic steatosis, as well as reduce body weight in a dose‐dependent manner. This may be because the nanoemulsion system diminishes the direct contact of capsaicin with the gastric mucosa and simultaneously improves the bioavailability of capsaicin.

### 7.8 Celastrol

Pentacyclic triterpene celastrol (CLT) is extracted from the root bark of *Tripterygium wilfordii* and has positive effects on alleviating inflammation, oxidative stress, and autoimmunity.^[^ ^190^ ^]^ Moreover, CLT may induce weight loss by suppressing lipid absorption and lipogenesis, as well as increasing lipolysis and thermogenesis.^[^ ^191^ ^]^ At the molecular level, CLT covalently inhibits the chaperone activity of 78 kDa glucose‐regulated protein, thereby disconnecting the transduction of endoplasmic reticulum stress signals to downstream inflammatory responses and lipid metabolism. However, the pharmaceutical and nutraceutical development of CLT is severely hampered by its limited bioavailability and potential toxicity. Overdose of CLT has been reported to cause cardiotoxicity, nephrotoxicity, and hepatotoxicity.^[^ ^192^ ^]^

The entrapment of CLT in NP possesses great value in overcoming the low water solubility, poor permeability, and off‐target side effects of CLT.^[^ ^193^ ^]^ CLT was loaded into poly(ethylene glycol)‐poly(ε‐caprolactone) copolymer (PEG‐PCL) nanomicelles via PEG‐PCL self‐assembly in oil‐in‐water emulsion, which results in nanocelastrol formulations (Figure 2L) that exhibited delayed time to blood peak and reduced clearance in mice.^[^ ^194^ ^]^ Comparative experiments were conducted to assess the antiobesity and anti‐inflammatory properties of nanocelastrol and CLT in HFD‐induced obese mice. The results showed that nanocelastrol was as effective as CLT in reducing body weight, fat mass, and glucose tolerance, as well as improving insulin sensitivity. In particular, a high dosage (7.5 mg kg^−1^ day^−1^ of CLT) of nanocelastrol could reduce body weight and fat mass in obese mice to the levels of mice fed a normal diet. Additionally, nanocelastrol significantly suppressed proinflammatory M1 macrophage polarization and marginally increased anti‐inflammatory M2 macrophage polarization. Regarding the safety of nanocelastrol, pathological analyses did not reveal that it led to toxic impacts on major organs, including heart, lungs, liver, and kidneys. Moreover, it did not induce any signs of anus inflammation and provided better protection against HFD‐evoked cell membrane disruption in comparison with CLT.

## 8 Challenges and Perspectives

**Figure 3:**
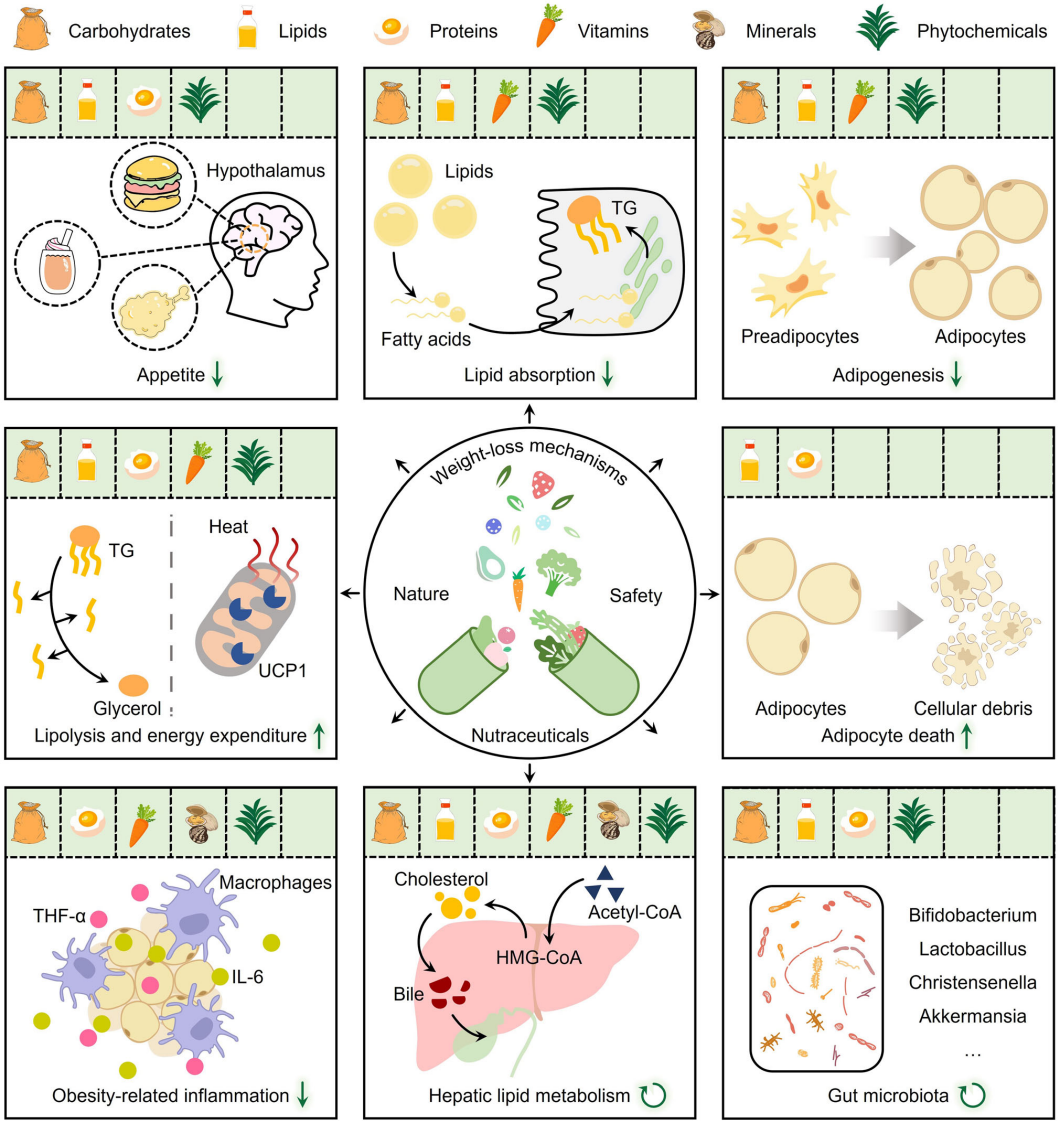
Overview of weight‐loss mechanisms of nutraceuticals. Downward green arrows denote inhibition. Upward green arrows denote enhancement. Rotating green arrows denote regulation.

**Figure 4:**
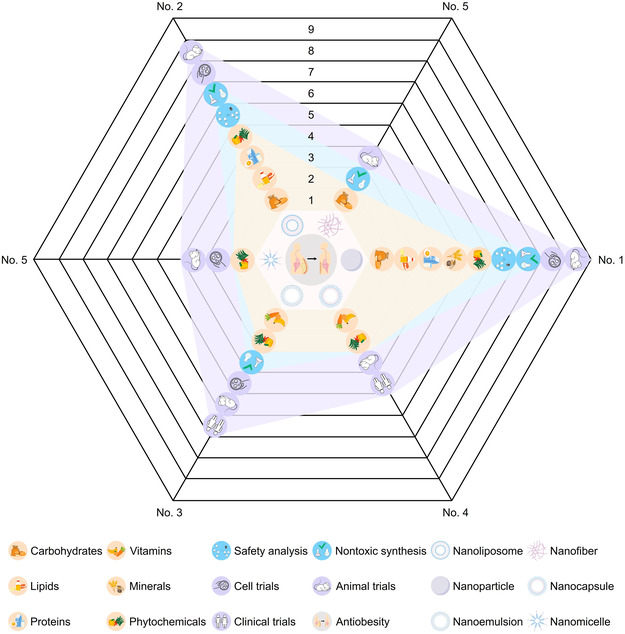
Potentials of six nanotechnologies in advancing nutraceutical applications for weight loss. Potential analyses encompass nutraceutical suitability, technical safety, and research completeness. Nutraceutical suitability refers to the categories of weight‐loss nutraceuticals (carbohydrates, lipids, proteins, vitamins, minerals, and phytochemicals) that can be modified by a specific nanotechnology. One point is awarded for each category. Technical safety includes two criteria: whether nontoxic synthesis is used and whether biosafety assessments are implemented. One point is awarded for each criterion met. Research completeness is assessed by whether research subjects involve cells, animals, and human populations. One point is awarded for each subject involved. Based on the total scores from all evaluations, six nanotechnologies are ranked from highest to lowest in terms of application potential and labeled as “no. 1–6.” Note: Those with the same score are ranked equally.

Unlike orthodox approaches, taking nutraceuticals is a straightforward, affordable, and relatively innocuous strategy for managing obesity. These substances can reduce body weight via multiple mechanisms, which encompass suppressing appetite, lipid absorption, and adipogenesis, increasing lipolysis and energy expenditure, inducing adipocyte and adipose endotheliocyte death, alleviating obesity‐related inflammation, as well as regulating hepatic lipid metabolism and gut microbiota (**Figure** **3**). Thanks to the burgeoning development of nanotechnology, low oral bioavailability, the core obstacle hindering the practical translational application of nutraceuticals, is expected to be effectively overcome. Here, we further semi‐quantitatively assess the application potentials of antiobesity nutraceuticals in six nanoformulations: NP, NL, nanofibers, nanoemulsions, nanocapsules, and nanomicelles. This assessment is based on nutraceutical suitability, technical safety, and research completeness (from cells and animals to humans). As illustrated in **Figure** **4**, NP rank first for their versatility, favorable safety characteristics, and relatively high research integrity, followed by NL, nanoemulsions, and nanocapsules. The last place is occupied by nanofibers and nanomicelles, whose applications are currently limited.

Although preclinical studies have demonstrated that most nanonutraceuticals have excellent weight‐loss capacities, safety and human effectiveness are still two primary challenges on their path to translation. Currently available slimming pharmaceuticals are often accompanied by safety concerns. Even FDA‐approved medications, such as well‐established Orlistat and spotlighted Semaglutide, have been linked to various negative sequelae, including nausea, vomiting, diarrhea, constipation, and liver injury.^[^ ^195^ ^]^ In contrast, nutraceuticals hold inherent safety advantages due to their natural origins. However, their nanomodifications may pose unforeseen health risks. A randomized clinical trial^[^ ^196^ ^]^ indicated that taking nanonutraceuticals for 1 year did not cause serious adverse events or related deaths, but a minority of participants experienced pruritus, muscle fasciculation, and mild hepatic function abnormalities. Crosslinkers, emulsifiers, and surfactants in the preparation of certain NP are capable of eliciting hepatotoxicity, nephrotoxicity, and other adverse reactions.^[^ ^197^, ^198^, ^199^ ^]^ Additionally, nanosized and free nutraceuticals have markedly distinct pharmacokinetics, pharmacodynamics, and biodistribution in vivo. NP larger than 5 nm can evade rapid renal clearance, resulting in prolonged circulation in the bloodstream.^[^ ^200^ ^]^ Those exceeding 200 nm are predominantly sequestered by hepatic tissues^[^ ^201^ ^]^ while smaller particles tend to distribute widely throughout the body. These size‐dependent properties may lead to the accumulation of nanonutraceuticals in nontarget organs, which engenders unintended side effects. Therefore, future research should focus on systematically evaluating the metabolic fate, nonspecific tissue toxicity, and long‐term safety of nanonutraceuticals to guide their rational design and synthesis with more biocompatible and smart materials.

In addition to safety issues, the effectiveness of antiobesity nanonutraceuticals on humans is nebulous at present. This may be related to the scarcity of clinical trials, but it seems particularly critical to maximize the weight‐loss potency of nanonutraceuticals without relying on dose effects. As most nanonutraceuticals are administered orally, the biggest difficulty for their clinical efficacy lies in protecting them from gastrointestinal digestion while ensuring their intact absorption into the bloodstream and precise delivery to target organs. This difficulty may be circumvented by optimizing their physicochemical properties (e.g., particle size, surface charge, and stability) and employing surface functionalization strategies^[^ ^202^, ^203^ ^]^ (e.g., fat targeting and sustained release). Moreover, tackling obesity requires a multifaceted approach, since its occurrence and maintenance involve intricate cell signaling pathways. Thus, the simultaneous nanodelivery of multiple nutraceuticals can contribute to a synergistic fight against obesity.^[^ ^204^ ^]^ It is worth attempting to ascertain characteristics of different combinations of nutraceuticals and assess the nature of interactions among them.

## 9 Summary

Specific nutrients are inextricably linked to the onset and progression of obesity. Their moderate supplementation can reverse obesity to some extent. However, the low water solubility, chemical instability, and high biodegradability of related nutraceuticals often result in their inability to be absorbed in large quantities by the digestive system or bioactivity loss before reaching target cells or organs. This poor bioavailability impedes their antiobesity actions. Optimistically, nanotechnology opens up an extremely promising avenue for surmounting these challenges. In this review, we summarize six major categories of nanonutraceuticals that can be used to fight obesity, detailing their characteristics, improved bioavailability, and weight‐loss efficacy. More in‐depth, we discuss their clinical application perspectives, challenges, and possible solutions. As a pivotal resource, this review can serve researchers and healthcare professionals working to prevent or treat obesity with nanonutraceuticals. It is highly anticipated that through their concerted efforts, the global obesity pandemic can be effectively curbed in an acceptable nutritional way.

## Conflict of Interest

The authors declare no conflict of interest.

## References

[1] N. H. Phelps , R. K. Singleton , B. Zhou , R. A. Heap , A. Mishra , J. E. Bennett , C. J. Paciorek , V. P. Lhoste , R. M. Carrillo‐Larco , G. A. Stevens , A. Rodriguez‐Martinez , Lancet 2024, 403, 1027.38432237

[2] M. Ng , E. Gakidou , J. Lo , Y. H. Abate , C. Abbafati , N. Abbas , M. Abbasian , A. Azargoonjahromi , Lancet 2025, 405, 813.40049186

[3] X. Jin , T. Qiu , L. Li , R. Yu , X. Chen , C. Li , C. G. Proud , T. Jiang , Acta Pharm. Sin. B 2023, 13, 2403. 37425065 10.1016/j.apsb.2023.01.012 PMC10326265

[4] A. Okunogbe , R. Nugent , G. Spencer , J. Powis , J. Ralston , J. Wilding , BMJ Glob. Health 2022, 7, e009773. 10.1136/bmjgh-2022-009773 PMC9494015 36130777

[5] K. H. Lewis , C. E. Sloan , D. H. Bessesen , D. Arterburn , BMJ 2024, 384, e072686. 38527759 10.1136/bmj-2022-072686

[6] U. F. Shaik Mohamed Sayed , S. Moshawih , H. P. Goh , N. Kifli , G. Gupta , S. K. Singh , D. K. Chellappan , K. Dua , A. Hermansyah , H. L. Ser , L.C. Ming , Front. Pharmacol. 2023, 14, 1182937. 37408757 10.3389/fphar.2023.1182937 PMC10318930

[7] S. B. Heymsfield , T. A. Wadden , N. Engl. J. Med. 2017, 376, 254. 28099824 10.1056/NEJMra1514009

[8] M. Watanabe , R. Risi , D. Masi , A. Caputi , A. Balena , G. Rossini , D. Tuccinardi , S. Mariani , S. Basciani , S. Manfrini , L. Gnessi , Nutrients 2020, 12, 2873. 32962190 10.3390/nu12092873 PMC7551574

[9] I. Dini , A. Mancusi , Molecules 2023, 28, 5357. 37513229 10.3390/molecules28145357 PMC10384751

[10] C. Dima , E. Assadpour , S. Dima , S. M. Jafari , Compr. Rev. Food Sci. Food Saf. 2020, 19, 954. 33331687 10.1111/1541-4337.12547

[11] D. J. McClements , S. M. Jafari , Adv. Colloid Interface Sci. 2018, 251, 55. 29248154 10.1016/j.cis.2017.12.001

[12] S. Gorantla , G. Wadhwa , S. Jain , S. Sankar , K. Nuwal , A. Mahmood , S. K. Dubey , R. Taliyan , P. Kesharwani , G. Singhvi , Drug Delivery Transl. Res. 2022, 12, 2359. 10.1007/s13346-021-01097-z 34845678

[13] X. Yao , B. Yang , J. Xu , Q. He , W. Yang , VIEW 2022, 3, 20200185.

[14] J. Guo , Y. Ping , H. Ejima , K. Alt , M. Meissner , J. J. Richardson , Y. Yan , K. Peter, D. von Elverfeldt, C. E. Hagemeyer, F. Caruso, Angew. Chem., Int. Ed. 2014, 53, 5546. 10.1002/anie.201311136 24700671

[15] H. Kaya‐Celiker , K. Mallikarjunan , Food Eng. Rev. 2012, 4, 114.

[16] Q. Zhao , S. Wang , Z. Lv , A. Zupanic , S. Guo , Q. Zhao , L. Jiang , Y. Yu, Biotechnol. Adv. 2022, 59, 107982. 35577226 10.1016/j.biotechadv.2022.107982

[17] R. K. Paul , P. Kesharwani , K. Raza , J. Biomater , Sci. Polym. Ed. 2021, 32, 2046. 10.1080/09205063.2021.1952381 34228585

[18] V. Singh , P. Kesharwani , J. Biomater , Sci. Polym. Ed. 2021, 32, 1882. 10.1080/09205063.2021.1938859 34078252

[19] A. A. Date , J. Hanes , L. M. Ensign , J. Controlled Release 2016, 240, 504. 10.1016/j.jconrel.2016.06.016 PMC5064878 27292178

[20] P. Kesharwani , V. Gajbhiye , N. K. Jain , Biomaterials 2012, 33, 7138. 22796160 10.1016/j.biomaterials.2012.06.068

[21] A.‐L. Schäfer , A. Eichhorst , C. Hentze , A. N. Kraemer , A. Amend , D. T. L. Sprenger , C. Fluhr , S. Finzel , C. Daniel , U. Salzer , M. Rizzi , Front. Immunol. 2021, 12, 696810. 34335609 10.3389/fimmu.2021.696810 PMC8320762

[22] S. Mayengbam , J. E. Lambert , J. A. Parnell , J. M. Tunnicliffe , A. C. Nicolucci , J. Han , T. Sturzenegger , J. Shearer , B. Mickiewicz , H. J. Vogel , K. L. Madsen , J. Nutr. Biochem. 2019, 64, 228. 30572270 10.1016/j.jnutbio.2018.11.003

[23] J. W. Anderson , P. Baird , R. H. Davis , S. Ferreri , M. Knudtson , A. Koraym , V. Waters , C. L. Williams , Nutr. Rev. 2009, 67, 188. 19335713 10.1111/j.1753-4887.2009.00189.x

[24] L. Wang , Q. Yang , Y. Yang , K. Luo , R. Bai , Int. J. Smart Nano Mater. 2023, 14, 57.

[25] H. Lu , Y. Gui , L. Zheng , X. Liu , Food Res. Int. 2013, 50, 121.

[26] Y.‐J. Lin , J. A. Shatkin , F. Kong , Carbohydr. Polym. 2019, 210, 157. 30732748 10.1016/j.carbpol.2019.01.029

[27] G. M. DeLoid , I. S. Sohal , L. R. Lorente , R. M. Molina , G. Pyrgiotakis , A. Stevanovic , R. Zhang , D. J. McClements , N. K. Geitner , D. W. Bousfield , K. W. Ng , ACS Nano 2018, 12, 6469. 29874029 10.1021/acsnano.8b03074 PMC6535802

[28] P. Thomas , T. Duolikun , N. P. Rumjit , S. Moosavi , C. W. Lai , M. R. Bin Johan , L. B. Fen , J. Mech. Behav. Biomed. Mater. 2020, 110, 103884. 32957191 10.1016/j.jmbbm.2020.103884

[29] Y. Habibi , L. A. Lucia , O. J. Rojas , Chem. Rev. 2010, 110, 3479. 20201500 10.1021/cr900339w

[30] M. S. M. E. Abdelbaky , H. S. Ibrahim , M. L. Hassan , Z. E. Sayed , ARC J. Nutr. Growth 2016, 2, 1.

[31] H. Lu , Y. Gui , T. Guo , Q. Wang , X. Liu , Food Funct. 2015, 6, 1185. 25710810 10.1039/c4fo00799a

[32] T. Yi , H. Zhao , Q. Mo , D. Pan , Y. Liu , L. Huang , H. Xu , B. Hu , H. Song , Materials 2020, 13, 5062. 33182719 10.3390/ma13225062 PMC7697919

[33] T. Nagano , H. Yano , Biosci. Biotechnol. Biochem. 2020, 84, 613. 31718523 10.1080/09168451.2019.1690975

[34] T. Nagano , H. Yano , Bioact. Carbohydr. Diet. Fibre 2020, 22, 100214.

[35] S. Gorgieva , Processes 2020, 8, 624.

[36] X. Zhai , D. Lin , Y. Zhao , W. Li , X. Yang , Food Funct. 2018, 9, 5260. 30238111 10.1039/c8fo01211c

[37] X. Zhai , D. Lin , Y. Zhao , W. Li , X. Yang , J. Agric. Food Chem. 2018, 66, 12706. 30411889 10.1021/acs.jafc.8b05036

[38] J. Huang , Q. Wu , Z. Lin , S. Liu , Q. Su , Y. Pan , Acta Sci. Pol. Technol. Aliment. 2020, 19, 279. 32978911 10.17306/J.AFS.0775

[39] M. V. Tzoumaki , T. Moschakis , E. Scholten , C. G. Biliaderis , Food Funct. 2013, 4, 121. 23064096 10.1039/c2fo30129f

[40] W. Ye , L. Liu , J. Yu , S. Liu , Q. Yong , Y. Fan , Food Nutr. Res. 2018, 62, 1295. 10.29219/fnr.v62.1295 PMC6052507 30038555

[41] M. Anraku , R. Tabuchi , S. Ifuku , T. Nagae , D. Iohara , H. Tomida , K. Uekama , T. Maruyama , S. Miyamura , F. Hirayama , M. Otagiri , Carbohydr. Polym. 2017, 161, 21. 28189231 10.1016/j.carbpol.2016.12.057

[42] K. Azuma , T. Nagae , T. Nagai , H. Izawa , M. Morimoto , Y. Murahata , T. Osaki , T. Tsuka , T. Imagawa , N. Ito , Y. Okamoto , Int. J. Mol. Sci. 2015, 16, 17445. 26263969 10.3390/ijms160817445 PMC4581201

[43] M. Goto , D. Iohara , A. Michihara , S. Ifuku , K. Azuma , D. Kadowaki , T. Maruyama , M. Otagiri , F. Hirayama , M. Anraku , Int. J. Biol. Macromol. 2020, 164, 659. 32698063 10.1016/j.ijbiomac.2020.07.184

[44] T. Kean , M. Thanou , Adv. Drug Delivery Rev. 2010, 62, 3. 10.1016/j.addr.2009.09.004 19800377

[45] W. L. Baker , A. Tercius , M. Anglade , C. M. White , C. I. Coleman , Ann. Nutr. Metab. 2009, 55, 368. 19923803 10.1159/000258633

[46] B. Shagdarova , M. Konovalova , V. Varlamov , E. Svirshchevskaya , Anti‐ObesityPolymers 2023, 15, 3967. 10.3390/polym15193967 PMC10575173 37836016

[47] A. M. Neyrinck , L. B. Bindels , F. De Backer , B. D. Pachikian , P. D. Cani , N. M. Delzenne , Int. Immunopharmacol. 2009, 9, 767. 19286482 10.1016/j.intimp.2009.02.015

[48] D. Xiao , W. Ren , P. Bin , S. Chen , J. Yin , W. Gao , G. Liu , Z. Nan , X. Hu , J. He , J. Funct. Foods 2016, 22, 166.

[49] N. S. Tapola , M. L. Lyyra , R. M. Kolehmainen , E. S. Sarkkinen , A. G. Schauss , J. Am. Coll. Nutr. 2008, 27, 22. 18460478 10.1080/07315724.2008.10719671

[50] M. Sumiyoshi , Y. Kimura , J. Pharm. Pharmacol. 2006, 58, 201. 16451748 10.1211/jpp.58.2.0007

[51] H. G. Choi , J. K. Kim , D. H. Kwak , J. R. Cho , J. Y. Kim , B. J. Kim , K. Y. Jung , B. K. Choi , M. K. Shin , Y. K. Choo , Arch. Pharm. Res. 2002, 25, 178. 12009032 10.1007/BF02976560

[52] H. Zhang , X. Zhong , Y. Tao , S. Wu , Z. Su , Int. J. Nanomed. 2012, 7, 4069. 10.2147/IJN.S33830 PMC3414085 22888243

[53] Y. Tao , H. Zhang , B. Gao , J. Guo , Y. Hu , Z. Su , J. Nanomater. 2011, 2011, 814606.

[54] H. Zhang , Y. Tao , J. Guo , Y. Hu , Z. Su , Int. Immunopharmacol. 2011, 11, 457. 21215349 10.1016/j.intimp.2010.12.015

[55] D. J. Machate , P. S. Figueiredo , G. Marcelino , R. C. A. Guimarães , P. A. Hiane , D. Bogo , V. A. Z. Pinheiro , L. C. S. D. Oliveira , A. Pott , Int. J. Mol. Sci. 2020, 21, 4093. 32521778 10.3390/ijms21114093 PMC7312778

[56] K. González‐Becerra , O. Ramos‐Lopez , E. Barrón‐Cabrera , J. I. Riezu‐Boj , F. I. Milagro , E. Martínez‐López , J. A. Martínez , Lipids Health Dis. 2019, 18, 178. 31615571 10.1186/s12944-019-1120-6 PMC6792183

[57] A. A. Papamandjaris , D. E. Macdougall , P. J. H. Jones , Life Sci. 1998, 62, 1203. 9570335 10.1016/s0024-3205(97)01143-0

[58] P. Schönfeld , L. Wojtczak , J. Lipid Res. 2016, 57, 943. 27080715 10.1194/jlr.R067629 PMC4878196

[59] S. A. Rial , A. D. Karelis , K.‐F. Bergeron , C. Mounier , Nutrients 2016, 8, 281. 27187452 10.3390/nu8050281 PMC4882694

[60] M.‐P. St‐Onge , B. Mayrsohn , M. O’Keeffe , H. R. Kissileff , A. R. Choudhury , B. Laferrère , Eur. J. Clin. Nutr. 2014, 68, 1134. 25074387 10.1038/ejcn.2014.145 PMC4192077

[61] M.‐P. St‐Onge , R. Ross , W. D. Parsons , P. J. H. Jones , Obes. Res. 2003, 11, 395. 12634436 10.1038/oby.2003.53

[62] J. Xia , P. Yu , Z. Zeng , M. Ma , X. Yan , J. Zhao , D. Gong , G. Zhang , J. Wang , Food Funct. 2022, 13, 8998. 35942878 10.1039/d2fo01711c

[63] Y. Zhang , Q. Xu , Y. Liu , X. Zhang , J. Wang , X. Yu , R. Zhang , X. U. E. Chao , X. Y. Yang , C. Y. Xue , Biomed. Environ. Sci. 2015, 28, 97. 25716560 10.3967/bes2015.012

[64] B. Marten , M. Pfeuffer , J. Schrezenmeir , Int. Dairy J. 2006, 16, 1374.

[65] W.‐L. Liu , W. Liu , C.‐M. Liu , S.‐B. Yang , J.‐H. Liu , H.‐J. Zheng , K.‐M. Su , Br. J. Nutr. 2011, 106, 1330. 21733323 10.1017/S0007114511002789 PMC3430869

[66] J.‐J. Li , C. J. Huang , D. Xie , Mol. Nutr. Food Res. 2008, 52, 631. 18306430 10.1002/mnfr.200700399

[67] O. Gudmundsen , H. Blankson , J. A. Stakkestad , H. Fagertun , E. Thom , J. Wadstein , J. Nutr. 2000, 130, 2943. 11110851 10.1093/jn/130.12.2943

[68] Y. Sun , X. Hou , L. Li , Y. Tang , M. Zheng , W. Zeng , X. Lei , Vet. Med. Sci. 2022, 8, 2538. 36104831 10.1002/vms3.921 PMC9677407

[69] A. Kennedy , K. Martinez , S. Schmidt , S. Mandrup , K. LaPoint , M. McIntosh , J. Nutr. Biochem. 2010, 21, 171. 19954947 10.1016/j.jnutbio.2009.08.003 PMC2826589

[70] D. Kim , J.‐H. Park , D.‐J. Kweon , G. D. Han , Int. J. Nanomed. 2013, 8, 451. 10.2147/IJN.S38430 PMC3575163 23429301

[71] L. Clément , H. Poirier , I. Niot , V. Bocher , M. Guerre‐Millo , S. Krief , B. Staels , P. Besnard , J. Lipid Res. 2002, 43, 1400. 12235171 10.1194/jlr.m20008-jlr200

[72] K. M. Mak , A. C. Shekhar , Anat. Rec. 2024, 307, 2162. 10.1002/ar.25333 37814787

[73] I. V. Maev , A. A. Samsonov , L. K. Palgova , C. S. Pavlov , E. I. Vovk , E. N. Shirokova , K. M. Starostin , BMJ Open Gastroenterol. 2020, 7, e000341. 10.1136/bmjgast-2019-000341 PMC7011021 32095253

[74] R. Amore , D. Amuso , V. Leonardi , F. Leva , A. C. Sibaud , A. Guida , E. Costa , F. Terranova , V. Amodeo , K. Gkritzalas , Plast. Reconstr. Surg. 2018, 6, e1794. 10.1097/GOX.0000000000001794 PMC6157955 30276043

[75] D. N. Reeds , B. S. Mohammed , S. Klein , C. B. Boswell , V. L. Young , Aesthet. Surg. J. 2013, 33, 400. 23439063 10.1177/1090820X13478630 PMC3667691

[76] Z. Kutlubay , J. Cosmet. Laser Ther. 2011, 13, 142. 21718184 10.3109/14764172.2011.594059

[77] S. M. Klein , S. Schreml , M. Nerlich , L. Prantl , Plast. Reconstr. Surg. 2009, 124, 419. 19644256 10.1097/PRS.0b013e3181adce61

[78] J. Janke , S. Engeli , K. Gorzelniak , F. C. Luft , J. Jordan , Obes. Facts 2009, 2, 36. 20054202 10.1159/000193461 PMC6444605

[79] L. Prantl , S. Gehmert , V. Brébant , V. Hoesl , Clin. Hemorheol. Microcirc. 2020, 75, 189. 31985455 10.3233/CH-190715

[80] F. Magkos , Rev. Endocr. Metab. Disord. 2020, 21, 329. 32740867 10.1007/s11154-020-09576-3

[81] M. Simonson , Y. Boirie , C. Guillet , Rev. Endocr. Metab. Disord. 2020, 21, 341. 32827096 10.1007/s11154-020-09574-5 PMC7455583

[82] Y.‐M. Kim , E.‐Y. Kim , I.‐H. Kim , T.‐J. Nam , Int. J. Mol. Med. 2015, 35, 1362. 25761066 10.3892/ijmm.2015.2127

[83] Y. Xiong , F.‐L. Zhang , J.‐R. Li , P.‐Z. Peng , B. Liu , L.‐N. Zhao , Food Biosci. 2022, 47, 101460.

[84] T. M. Allen , P. R. Cullis , Adv. Drug Delivery Rev. 2013, 65, 36. 10.1016/j.addr.2012.09.037 23036225

[85] S. Krobthong , Y. Yingchutrakul , W. Visessanguan , T. Mahatnirunkul , P. Samutrtai , C. Chaichana , P. Papan , K. Choowongkomon Foods 2021, 10, 2157. 34574267 10.3390/foods10092157 PMC8468392

[86] D.‐H. Kim , S. C. Woods , R. J. Seeley , Diabetes 2010, 59, 907. 20103704 10.2337/db09-1141 PMC2844838

[87] R. Kunitomo , Y. Miyauchi , M. Inoue , J. Biol. Chem. 1992, 267, 8732. 1315736

[88] M. N. Hossen , K. Kajimoto , H. Akita , M. Hyodo , H. Harashima , J. Controlled Release 2012, 163, 101. 10.1016/j.jconrel.2012.09.002 22982237

[89] M. N. Hossen , K. Kajimoto , H. Akita , M. Hyodo , T. Ishitsuka , H. Harashima , Mol. Ther. 2013, 21, 533. 23295953 10.1038/mt.2012.256 PMC3589155

[90] J. A. Do Nascimento Campos Ribeiro , A. C. Serquiz , P. F. Dos Santos Silva , P. B. B. M. Barbosa , T. B. M. Sampaio , R. F. De Araújo , A. S. De Oliveira , R. J. A. Machado , B. L. L. Maciel , A. F. Uchôa , E. A. D. Santos , Clinics 2015, 70, 136. 25789523 10.6061/clinics/2015(02)11 PMC4351314

[91] V. C. O. Lima , A. B. S. Luz , M. S. M. Amarante , M. C. J. S. Lima , F. M. C. Carvalho , J. B. S. Figueredo , P. P. A. Santos , F. V. L. Ladd, B. L. L. Maciel, A. F. Uchôa, A. H. A Morais, Obes. Facts 2021, 14, 357. 34256373 10.1159/000516548 PMC8406341

[92] F. M. C. Carvalho , V. C. O. Lima , I. S. Costa , A. F. Medeiros , A. C. Serquiz , M. C. J. S. Lima , R. P. Serquiz , B. L. Maciel , A. F. Uchôa , E. A. Santos , A. H. Morais , Nutrients 2016, 8, 544. 27690087 10.3390/nu8100544 PMC5083972

[93] A. F. Medeiros , I. S. Costa , F. M. C. Carvalho , S. Kiyota , B. B. P. Souza , D. N. Sifuentes , R. P. Serquiz , B. L. L. Maciel , A. F. Uchôa , E. A. D. Santos , A. H. D. A. Morais , J. Enzyme Inhib. Med. Chem. 2018, 33, 334. 29322840 10.1080/14756366.2017.1419220 PMC6010142

[94] J. L. C. De Queiroz , R. O. De Araújo Costa , L. L. Rodrigues Matias , A. F. De Medeiros , A. F. Teixeira Gomes , T. D. Santos Pais , T. S. Passos , B. L. L. Maciel , E. A. Dos Santos , A. H. D. A. Morais , Food Hydrocoll. 2018, 84, 247.

[95] R. O. A. Costa , L. L. R. Matias , T. S. Passos , J. L. C. de Queiroz , F. M. C. de Carvalho , B. L. L. Maciel , A. F. Uchôa , I. R. Amado , C. Gonçalves, L. Pastrana, A. H. A. Morais Future Foods 2020, 1–2, 100001.

[96] R. O. A. Costa , I. Medeiros , J. L. C. De Queiroz , L. L. R. Matias , M. S. R. Lima , G. S. D. Oliveira , A. J. F. C. Aguiar , I. S. Costa , E. M. D. S. Silva , N. C. S. Dos Santos , T. S. Passos , Foods 2022, 11, 3526.36360138

[97] C. Y. Lee , J. Nutr. Sci. 2023, 12, e47. 37123391 10.1017/jns.2023.30 PMC10131053

[98] C. Bertoncini‐Silva , J.‐M. Zingg , P. G. Fassini , V. M. M. Suen , BioFactors 2023, 49, 297. 36468445 10.1002/biof.1921

[99] W. S. Blaner , Pharmacol. Ther. 2019, 197, 153. 30703416 10.1016/j.pharmthera.2019.01.006 PMC6520171

[100] A. Carazo , K. Macáková , K. Matoušová , L. K. Krčmová , M. Protti , P. Mladěnka , Nutrients 2021, 13, 1703. 34069881 10.3390/nu13051703 PMC8157347

[101] Z. Huang , Y. Liu , G. Qi , D. Brand , S. G. Zheng , J. Clin. Med. 2018, 7, 258. 30200565 10.3390/jcm7090258 PMC6162863

[102] S. Thomas‐Valdés , M. G. V. Tostes , P. C. Anunciação , B. P. da Silva , H. M. P. Sant’Ana , Crit. Rev. Food Sci. Nutr. 2017, 57, 3332. 26745150 10.1080/10408398.2015.1117413

[103] C. C. Gomes , T. S. Passos , A. H. A. Morais , Nutrients 2021, 13, 1921. 34204998 10.3390/nu13061921 PMC8228342

[104] N. Yao , S. Yan , Y. Guo , H. Wang , X. Li , L. Wang , W. Hu , B. Li , W. Cui , Food Funct. 2021, 12, 4768. 33977977 10.1039/d1fo00004g

[105] H. Maeda , J. Oleo Sci. 2015, 64, 125. 25748372 10.5650/jos.ess14226

[106] M. A. Gammone , N. D’Orazio , Mar. Drugs 2015, 13, 2196. 25871295 10.3390/md13042196 PMC4413207

[107] T. Okada , Y. Mizuno , S. Sibayama , M. Hosokawa , K. Miyashita , J. Food Sci. 2011, 76, H2. 21535684 10.1111/j.1750-3841.2010.01878.x

[108] F. Saponaro , A. Saba , R. Zucchi , Int. J. Mol. Sci. 2020, 21, 6573. 32911795 10.3390/ijms21186573 PMC7554947

[109] J. C. Gallagher , C. J. Rosen , Lancet Diabetes Endocrinol. 2023, 11, 362.37004709

[110] M. Cojic , R. Kocic , A. Klisic , G. Kocic , Front. Endocrinol. 2021, 12, 610893. 10.3389/fendo.2021.610893 PMC8417320 34489860

[111] L. K. Almeida Moreira Leal , L. A. Lima , P. E. Alexandre de Aquino , J. A. Costa de Sousa , C. V. Jataí Gadelha , I. B. Felício Calou , M. J. Pereira Lopes , F. A. Viana Lima, K. R. Tavares Neves, G. Matos de Andrade, G. Socorro de Barros Viana, Eur. J. Pharmacol. 2020, 879, 173099. 32360837 10.1016/j.ejphar.2020.173099

[112] F. P. Reiter , L. Ye , F. Bösch , R. Wimmer , R. Artmann , A. Ziesch , V. Kanitz , D. Mayr , C. J. Steib , M. Trauner , I. Regel , Lab. Invest. 2019, 99, 1906. 31467426 10.1038/s41374-019-0310-1

[113] M. M. Cordeiro , P. B. Biscaia , J. Brunoski , R. A. Ribeiro , G. C. N. Franco , D. X. Scomparin , Life Sci. 2021, 278, 119550. 33932442 10.1016/j.lfs.2021.119550

[114] M. El‐Sherbiny , M. Eldosoky , M. El‐Shafey , G. Othman , H. A. Elkattawy , T. Bedir , N. M. Elsherbiny , Chem. Biol. Interact. 2018, 288, 65. 29653100 10.1016/j.cbi.2018.04.010

[115] P. Sharifan , A. Ziaee , S. Darroudi , M. Rezaie , M. Safarian , S. Eslami , M. Khadem‐Rezaiyan , M. Tayefi , M. Mohammadi Bajgiran , H. Ghazizadeh , Z. Khorasanchi , Curr. Med. Res. Opin. 2021, 37, 579. 33434080 10.1080/03007995.2021.1874324

[116] P. Sharifan , M. Rashidmayvan , Z. Khorasanchi , S. Darroudi , A. Heidari , F. Hoseinpoor , H. Vatanparast , M. Safarian , S. Eslami , A. Afshari , Z. Asadi , J. Health Popul. Nutr. 2022, 41, 8. 35236423 10.1186/s41043-022-00283-0 PMC8889656

[117] L. Wang , W. Liu , S. Bi , L. Zhou , L. Li , PLoS One 2023, 18, e0295765. 38150411 10.1371/journal.pone.0295765 PMC10752540

[118] R. M. Smita , A. P. R. Shuvo , S. Raihan , R. Jahan , F. A. Simin , A. Rahman , S. Biswas , L. Salem , M. A. T. Sagor Curr. Diabetes Rev. 2022, 18, e171121197987. 34789132 10.2174/1573399818666211117104626

[119] M. Maywald , I. Wessels , L. Rink , Int. J. Mol. Sci. 2017, 18, 2222. 29064429 10.3390/ijms18102222 PMC5666901

[120] A. B. G. Lansdown , U. Mirastschijski , N. Stubbs , E. Scanlon , M. S. Ågren , Wound Repair Regen. 2007, 15, 2. 17244314 10.1111/j.1524-475X.2006.00179.x

[121] P.‐H. Lin , M. Sermersheim , H. Li , P. H. U. Lee , S. M. Steinberg , J. Ma , Nutrients 2018, 10, 16. 10.3390/nu10010016 PMC5793244 29295546

[122] C. J. Frederickson , J.‐Y. Koh , A. I. Bush , Nat. Rev. Neurosci. 2005, 6, 449. 15891778 10.1038/nrn1671

[123] M. Kawahara , K. Tanaka , M. Kato‐Negishi , Nutrients 2018, 10, 147. 29382141 10.3390/nu10020147 PMC5852723

[124] S. R. Kimball , S.‐J. Chen , R. Risica , L. S. Jefferson , A. E. Leure‐dupree , Metabolism 1995, 44, 126. 7854157 10.1016/0026-0495(95)90299-6

[125] M. K. Mohammad , Z. Zhou , M. Cave , A. Barve , C. J. McClain , Nutr. Clin. Pract. 2012, 27, 8. 22307488 10.1177/0884533611433534 PMC6027651

[126] R. S. MacDonald , J. Nutr. 2000, 130, 1500S. 10801966 10.1093/jn/130.5.1500S

[127] D. D. N. Marreiro , M. Fisberg , S. M. F. Cozzolino , Biol. Trace Elem. Res. 2002, 86, 107. 12008974 10.1385/bter:86:2:107

[128] M. J. Rios‐Lugo , C. Madrigal‐Arellano , D. Gaytán‐Hernández , H. Hernández‐Mendoza , E. T. Romero‐Guzmán , Biol. Trace Elem. Res. 2020, 198, 51. 32020525 10.1007/s12011-020-02060-8

[129] L. Payahoo , A. Ostadrahimi , M. Mobasseri , Y. K. Bishak , N. Farrin , A. Jafarabadi , S. Mahluji , Adv. Pharm. Bull. 2013, 3, 161. 24312830 10.5681/apb.2013.027 PMC3846058

[130] J. Kim , J. Ahn , Biol. Trace Elem. Res. 2014, 157, 101. 24402636 10.1007/s12011-013-9885-3

[131] Y. Liu , S. Zong , J. Li , Appl. Biochem. Biotechnol. 2020, 190, 475. 31385191 10.1007/s12010-019-03115-w

[132] S. Dogra , A. K. Kar , K. Girdhar , P. V. Daniel , S. Chatterjee , A. Choubey , S. Ghosh , S. Patnaik , D. Ghosh , P. Mondal , Nanomed. Nanotechnol. Biol. Med. 2019, 17, 210. 10.1016/j.nano.2019.01.013 30708053

[133] W. Mertz , J. Nutr. 1993, 123, 626. 8463863 10.1093/jn/123.4.626

[134] J. B. Vincent , H. C. Lukaski , Adv. Nutr. 2018, 9, 505. 30032219 10.1093/advances/nmx021 PMC6054252

[135] C. Tsang , M. Taghizadeh , E. Aghabagheri , Z. Asemi , S. Jafarnejad , Clin. Obes. 2019, 9, e12313. 31115179 10.1111/cob.12313

[136] A. T. Hung , B. J. Leury , M. A. Sabin , F. Fahri , K. DiGiacomo , T.‐F. Lien , F. R. Dunshea , Animals 2020, 10, 1685. 32961883 10.3390/ani10091685 PMC7552722

[137] A. T. Hung , B. J. Leury , M. A. Sabin , T. F. Lien , F. R. Dunshea , Anim. Prod. Sci. 2015, 55, 454.

[138] H. Li , J. Qi , L. Li , Pharmacol. Res. 2019, 147, 104393. 31401211 10.1016/j.phrs.2019.104393

[139] E. Kim , S. Jeon , Nutrients 2023, 15, 703. 36771408 10.3390/nu15030703 PMC9920427

[140] J. Luo , Z. Yu , J. Tovar , A. Nilsson , B. Xu , Pharmacol. Res. 2022, 184, 106461. 36152739 10.1016/j.phrs.2022.106461

[141] X. Li , R. R. Holt , C. L. Keen , L. S. Morse , A. M. Zivkovic , G. Yiu , R. M. Hackman , Nutr. Rev. 2023, 81, 670. 36094616 10.1093/nutrit/nuac076 PMC11494239

[142] S. S. Gopal , S. M. Eligar , B. Vallikannan , G. Ponesakki , Biochim. Biophys. Acta BBA ‐ Mol. Cell Biol. Lipids 2021, 1866, 158812. 10.1016/j.bbalip.2020.158812 32920140

[143] S. S. Gopal , S. V. Sukhdeo , B. Vallikannan , G. Ponesakki , Phytother. Res. 2023, 37, 329. 36086831 10.1002/ptr.7615

[144] I. Bhat , U. G. Yathisha , I. Karunasagar , B. S. Mamatha , Nutr. Rev. 2020, 78, 709. 31925437 10.1093/nutrit/nuz096

[145] A. G. Murillo , D. Aguilar , G. H. Norris , D. M. DiMarco , A. Missimer , S. Hu , J. A. Smyth , S. Gannon , C. N. Blesso , Y. Luo , M. L Fernandez , J. Nutr. 2016, 146, 1961. 27581580 10.3945/jn.116.235374

[146] L.‐X. Zhang , C.‐X. Li , M. U. Kakar , M. S. Khan , P.‐F. Wu , R. M. Amir , D.‐F. Dai , M. Naveed , Q. Y. Li , M. Saeed , J. Q. Shen , Biomed. Pharmacother. 2021, 143, 112164. 34649335 10.1016/j.biopha.2021.112164

[147] X. Huang , X. Li , M. Xie , Z. Huang , Y. Huang , G. Wu , Z. Peng , Y. N. Sun , Q. L. Ming , Y. X. Liu , J. P. Chen , Chem. Biol. Interact. 2019, 306, 29. 30954463 10.1016/j.cbi.2019.04.001

[148] Z. Li , Z. Zhang , Y. Sun , W. Li , X. Feng , W. Zhu , C. Sifan Fed. Am. Soc. Exp. Biol. 2020, 34, 4527. 32003501 10.1096/fj.201902222R

[149] N. Drabińska , E. Jarocka‐Cyrta , Int. J. Mol. Sci. 2022, 23, 15279. 36499603 10.3390/ijms232315279 PMC9739931

[150] Y. Zu , H. Overby , G. Ren , Z. Fan , L. Zhao , S. Wang , Colloids Surf., B 2018, 164, 414. 10.1016/j.colsurfb.2017.12.044 PMC5915363 29433059

[151] S. Wan , L. Zhang , Y. Quan , K. Wei , R. Soc. Open Sci. 2018, 5, 181457. 30564426 10.1098/rsos.181457 PMC6281916

[152] M. Ahmad , A. Gani , Carbohydr. Polym. 2021, 251, 117111. 33142648 10.1016/j.carbpol.2020.117111

[153] M. Ahmad , A. Gani , Food Chem. 2021, 352, 129323. 33691210 10.1016/j.foodchem.2021.129323

[154] Y. Wu , Q. Ma , Q. Liu , M. Wang , W. Wei , G. Gong , Y. He , Y. Wang , Y. Zheng , L. Yang , G. Nyström , Cell Biomater. 2025, 1, 100019.

[155] W.‐F. Lai , M. M. F. A. Baig , W.‐T. Wong , B. T. Zhu , Trends Food Sci. Technol. 2020, 102, 271.

[156] S. Klaus , S. Pültz , C. Thöne‐Reineke , S. Wolfram , Int. J. Obes. 2005, 29, 615. 10.1038/sj.ijo.0802926 15738931

[157] J. Huang , Y. Zhang , Y. Zhou , Z. Zhang , Z. Xie , J. Zhang , X. Wan , J. Agric. Food Chem. 2013, 61, 8565. 23992224 10.1021/jf402004x

[158] I.‐J. Chen , C.‐Y. Liu , J.‐P. Chiu , C.‐H. Hsu , Clin. Nutr. 2016, 35, 592. 26093535 10.1016/j.clnu.2015.05.003

[159] J. D. Lambert , C. S. Yang , J. Nutr. 2003, 133, 3262S. 14519824 10.1093/jn/133.10.3262S

[160] B. Zhang , R. Yao , C. Hu , M. F. Maitz , H. Wu , K. Liu , L. Yang , R. Luo , Y. Wang , Biomaterials 2021, 269, 120418. 33143876 10.1016/j.biomaterials.2020.120418

[161] O. Krupkova , S. J. Ferguson , K. Wuertz‐Kozak , J. Nutr. Biochem. 2016, 37, 1. 27770867 10.1016/j.jnutbio.2016.01.002

[162] Z. Hong , Y. Xu , J.‐F. Yin , J. Jin , Y. Jiang , Q. Du , J. Agric. Food Chem. 2014, 62, 12603. 25483592 10.1021/jf504603n

[163] L. Montaldo , A. Gallo , G. Rocha , C. Csernoch , M. D. Marzi , L. N. Guerra , Ther. Delivery 2023, 14, 675. 10.4155/tde-2023-0018 38018449

[164] A. Smeriglio , D. Barreca , E. Bellocco , D. Trombetta , Phytother. Res. 2016, 30, 1265. 27221033 10.1002/ptr.5642

[165] B.‐H. Chen , B. Stephen Inbaraj , Nutrients 2019, 11, 1052. 31083417 10.3390/nu11051052 PMC6566753

[166] H. Song , X. Shen , R. Deng , Y. Zhang , X. Zheng , Nutrition 2021, 86, 111176. 33621858 10.1016/j.nut.2021.111176

[167] T. K. O. Rosales , J. P. Fabi , Colloids Surf., B 2022, 218, 112707. 10.1016/j.colsurfb.2022.112707 35907354

[168] P. R. Sreerekha , P. K. Dara , D. K. Vijayan , N. S. Chatterjee , M. Raghavankutty , S. Mathew , C. N. Ravishankar , R. Anandan , Carbohydr. Polym. Technol. Appl. 2021, 2, 100051.

[169] A. Di Petrillo , G. Orrù , A. Fais , M. C. Fantini , Phytother. Res. 2022, 36, 266. 34709675 10.1002/ptr.7309 PMC8662201

[170] P. Singh , Y. Arif , A. Bajguz , S. Hayat , Plant Physiol. Biochem. 2021, 166, 10. 34087741 10.1016/j.plaphy.2021.05.023

[171] M.‐J. Seo , Y.‐J. Lee , J.‐H. Hwang , K.‐J. Kim , B.‐Y. Lee , J. Nutr. Biochem. 2015, 26, 1308. 26277481 10.1016/j.jnutbio.2015.06.005

[172] J.‐S. Lee , Y.‐J. Cha , K.‐H. Lee , J.‐E. Yim , Nutr. Res. Pract. 2016, 10, 175. 27087901 10.4162/nrp.2016.10.2.175 PMC4819128

[173] A. Hosseini , B. M. Razavi , M. Banach , H. Hosseinzadeh , Phytother. Res. 2021, 35, 5352. 34101925 10.1002/ptr.7144

[174] P. Mukhopadhyay , S. Maity , S. Mandal , A. S. Chakraborti , A. K. Prajapati , P. P. Kundu , Carbohydr. Polym. 2018, 182, 42. 29279124 10.1016/j.carbpol.2017.10.098

[175] W. Zhang , C. Liang , H. Liu , Z. Li , R. Chen , M. Zhou , D. Li , Q. Ye , C. Luo , J. Sun , Asian J. Pharm. Sci. 2017, 12, 586. 32104372 10.1016/j.ajps.2017.08.003 PMC7032188

[176] J. Liu , J. Ethnopharmacol. 1995, 49, 57.8847885

[177] C. L. De Melo , M. G. R. Queiroz , S. G. C. Fonseca , A. M. C. Bizerra , T. L. G. Lemos , T. S. Melo , F. A. Santos , V. S. Rao , Chem. Biol. Interact. 2010, 185, 59. 20188082 10.1016/j.cbi.2010.02.028

[178] W. Li , H. Zeng , M. Xu , C. Huang , L. Tao , J. Li , T. Zhang , H. Chen , J. Xia , C. Li , X. Li , Front. Pharmacol. 2021, 12, 697483. 34393781 10.3389/fphar.2021.697483 PMC8361479

[179] F. Z. Djeziri , M. Belarbi , B. Murtaza , A. Hichami , C. Benammar , N. A. Khan , Biochimie 2018, 152, 110. 29966735 10.1016/j.biochi.2018.06.025

[180] S. Wang , L.‐B. Du , L. Jin , Z. Wang , J. Peng , N. Liao , Y.‐Y. Zhao , J. L. Zhang , J. Pauluhn , C. X. Hai , X. Wang , Biomed. Pharmacother. 2018, 108, 1181. 30372819 10.1016/j.biopha.2018.09.150

[181] P. Anand , R. Privitera , P. Donatien , H. Fadavi , S. Tesfaye , V. Bravis , V. P. Misra , Front. Neurol. 2022, 13, 998904. 36388188 10.3389/fneur.2022.998904 PMC9643187

[182] Q. Zhang , P. Luo , F. Xia , H. Tang , J. Chen , J. Zhang , D. Liu , Y. Zhu , Y. Liu , L. Gu , L. Zheng , Cell Chem. Biol. 2022, 29, 1248. 35858615 10.1016/j.chembiol.2022.06.011

[183] M. Chen , C. Xiao , W. Jiang , W. Yang , Q. Qin , Q. Tan , B. Lian , C. Wei, Drug Des. Devel. Ther. 2021, 15, 125. 10.2147/DDDT.S269901 PMC7811378 33469265

[184] A. Szallasi , Biomolecules 2022, 12, 1783.36551210

[185] R. Li , Y. Lan , C. Chen , Y. Cao , Q. Huang , C.‐T. Ho , M. Lu , Food Funct. 2020, 11, 7356. 32820787 10.1039/d0fo01467b

[186] A. Rezazadeh , H. Hamishehkar , A. Ehsani , Z. Ghasempour , E. Moghaddas Kia , Crit. Rev. Food Sci. Nutr. 2023, 63, 4009. 34751073 10.1080/10408398.2021.1997904

[187] Y. Feng , Y. Zhu , J. Wan , X. Yang , C. K. Firempong , J. Yu , X. Xu , J. Funct. Foods 2018, 44, 137.

[188] J.‐Y. Kim , M.‐S. Lee , S. Jung , H. Joo , C.‐T. Kim , I.‐H. Kim , S. Seo , S. Oh , Y. Kim , Int. J. Nanomedicine 2014, 9, 301. 24403834 10.2147/IJN.S52414 PMC3883587

[189] M. Lu , Y. Cao , C.‐T. Ho , Q. Huang , Food Funct. 2017, 8, 1803. 28443906 10.1039/c7fo00173h

[190] S. W. Ng , Y. Chan , D. K. Chellappan , T. Madheswaran , F. Zeeshan , Y. L. Chan , T. Collet , T. Collet , G. Gupta , B. G. Oliver , P. Wark , N. Hansbro , Biomed. Pharmacother. 2019, 109, 1785. 30551432 10.1016/j.biopha.2018.11.051

[191] D. Luo , N. Fan , X. Zhang , F. Y. Ngo , J. Zhao , W. Zhao , M. Huang , D. Li , Y. Wang , J. Rong , eLife 2022, 11, e72182. 35138251 10.7554/eLife.72182 PMC8828050

[192] J. Song , G.‐N. He , L. Dai , Biomed. Pharmacother. 2023, 162, 114705. 37062220 10.1016/j.biopha.2023.114705

[193] L. Guo , Y. Zhang , K. T. Al‐Jamal , Biomater. Sci. 2021, 9, 6355. 34582530 10.1039/d1bm00639h

[194] J. Zhao , D. Luo , Z. Zhang , N. Fan , Y. Wang , H. Nie , J. Rong , J. Controlled Release 2019, 310, 188. 10.1016/j.jconrel.2019.08.026 31454532

[195] T. D. Müller , M. Blüher , M. H. Tschöp , R. D. DiMarchi , Nat. Rev. Drug Discovery 2022, 21, 201. 34815532 10.1038/s41573-021-00337-8 PMC8609996

[196] M. Ahmadi , E. Agah , S. Nafissi , M. R. Jaafari , M. H. Harirchian , P. Sarraf , S. Faghihi‐Kashani , S. J. Hosseini , A. Ghoreishi , V. Aghamollaii , M. Hosseini , Neurotherapeutics 2018, 15, 430. 29352425 10.1007/s13311-018-0606-7 PMC5935637

[197] C. Kang , J. Wang , R. Li , J. Gong , K. Wang , Y. Wang , Z. Wang , R. He , F. Li , Molecules 2023, 28, 5955.37630208

[198] X. Liu , W. Wang , Q. Li , H. Niu , W. Zhang , Int. J. Smart Nano Mater. 2024, 15, 610.

[199] V. Vilas‐Boas , M. Vinken , Arch. Toxicol. 2021, 95, 27. 33155068 10.1007/s00204-020-02940-x

[200] W. Zhang , A. Mehta , Z. Tong , L. Esser , N. H. Voelcker , Adv. Sci. 2021, 8, 2003937. 10.1002/advs.202003937 PMC8132167 34026447

[201] Q. Chen , L. Yuan , W.‐C. Chou , Y.‐H. Cheng , C. He , N. A. Monteiro‐Riviere , J. E. Riviere , Z. Lin , ACS Nano 2023, 17, 19810. 37812732 10.1021/acsnano.3c04037 PMC10604101

[202] J. Pan , H. Liao , G. Gong , Y. He , Q. Wang , L. Qin , Y. Zhang , H. Ejima , B. L. Tardy , J. J. Richardson , J. Shang ,J. Controlled Release 2023, 360, 433. 10.1016/j.jconrel.2023.07.003 37422124

[203] T. Liang , Z. Xing , L. Jiang , J.‐J. Zhu , VIEW 2021, 2, 20200131.

[204] A. Sarkar , H. Li , D. Cray , S. Boxall , Food Hydrocoll. 2018, 77, 436.

